# Skin barrier‐related genes in childhood atopic dermatitis, asthma, and allergy: A systematic review and meta‐analysis

**DOI:** 10.1111/pai.70326

**Published:** 2026-04-23

**Authors:** I. Husain, K. Patel, C. Holden, L. Jervis, K. Barnard, E. Youssef, J. Felton, S. Bremner, S. J. Brown, S. Mukhopadhyay

**Affiliations:** ^1^ Department of Clinical and Experimental Medicine Brighton and Sussex Medical School Brighton UK; ^2^ Royal Alexandra Children's Hospital University Hospitals Sussex NHS Trust Brighton UK; ^3^ Royal Free London NHS Foundation Trust London UK; ^4^ Department of Medical Education Brighton and Sussex Medical School Brighton UK; ^5^ Croydon Health Services NHS Trust London UK; ^6^ Division of Methodologies, Faculty of Nursing, Midwifery and Palliative Care King's College London London UK; ^7^ Centre for Genomic and Experimental Medicine Institute of Genetics and Cancer, University of Edinburgh Edinburgh UK

**Keywords:** asthma, atopic dermatitis, food allergy, genetics, skin‐barrier

## Abstract

Atopic dermatitis (AD), asthma, food allergy, and respiratory allergy are common childhood conditions with significant disease burden. Current understanding implicates the skin‐barrier and the dual‐allergen hypotheses in the pathophysiology of these allergic conditions. This systematic review and meta‐analysis aimed to investigate the role of genes associated with primary skin barrier dysfunction in childhood atopic conditions. We conducted a comprehensive search across Embase, MEDLINE, Emcare, CINAHL, and Cochrane Central databases, yielding 6018 abstracts and 941 full‐text articles, of which 60 met the inclusion criteria. Quality assessment was performed using the Q‐GENIE tool. The full protocol is available on the PROSPERO database (registration ID CRD42022355771). Our analysis confirms a role for the filaggrin (FLG) gene, with meta‐analysis revealing associations between FLG loss‐of‐function (LOF) mutations and AD (OR 2.426, 95% CI 1.890–3.114) in cohort studies and (OR 4.44, 95% CI 2.42–8.12) in case–control studies, asthma (OR 1.90, 95% CI 1.33–2.71), and food allergy (OR 1.79, 95% CI 1.11–2.88) in cohort studies. No statistically significant associations were found between FLG mutations and food allergy in case–control studies, or respiratory allergies. Included studies with insufficient clinical homogeneity for meta‐analysis were presented as narrative synthesis, with studies looking at genetic associations with *FLG* (*n* = 28), FLG‐2 (*n* = 1) and IL‐18 (*n* = 1). The findings underscore the critical role of *FLG* mutations in childhood allergic conditions, particularly AD and asthma. However, we did not identify quantitative evidence for a clinically significant role for other skin barrier‐related genes in relation to childhood allergy. Limitations include the exclusion of non‐English‐language studies and potential omissions of genes not represented in the Gene Ontology Skin Barrier database. Future research could explore personalized medicine according to *FLG* genotype to mitigate the effects of environmental exposures and reduce the burden of atopic diseases in children.

**Key message**

This systematic review and meta‐analysis aims to define current knowledge on primary skin barrier‐related genetic mutations and their role in the pathogenesis of childhood atopic disease. The identification of genes implicated in skin barrier function allows for further characterization of their roles in the incidence and severity of childhood eczema, allergy, and asthma. Further research exploring the interplay between genetic and environmental factors should aim to explore simple lifestyle changes which may be of use in families of children with primary skin barrier defects. This may, in turn, reduce the burden of childhood atopic disease.

## 1 BACKGROUND

Atopic dermatitis (AD) is a chronic skin condition characterized by persistent dryness and periods of pruritus and inflamed skin.^1^, ^2^, ^3^ AD commonly develops in childhood, often first presenting in infancy, but may occur at any age^4^, ^5^, ^6^ and may fluctuate throughout life.^6^

According to studies in children with early onset severe AD, over 60% develop asthma before the age of 7, over 40% develop IgE mediated food allergies, and similar cohorts show a 3‐fold increased risk of rhinitis.^7^ Some have termed this course the ‘the atopic march’, but the trajectory is unpredictable and therefore the term has been criticized.^8^ It is estimated that 40%–50% of children in the United Kingdom (UK) have been diagnosed with at least one allergic condition, contributing to a high childhood atopic disease burden.^9^, ^10^

Disruption of the epithelial barrier of the skin, dysbiosis of the gut microbiome, increased oxidative stress and epigenetic changes^11^, ^12^ may contribute to the co‐existence of atopic disorders. The skin acts as a barrier, comprising corneocytes, Langerhans cells, keratinocytes and lipids, which are joined together by adherens junctions, tight junctions and desmosomes^13^; this barrier protects the body from physical, chemical, and immunological stimuli.^14^ The skin barrier hypothesis of atopic disease proposes that, if the skin barrier is impaired in certain individuals, the skin will be more permeable to environmental stimuli, including allergens, generating an inflammatory response within the skin which can progress to AD.^15^, ^16^ The dual‐allergen exposure hypothesis builds upon this by suggesting that exposure to certain stimuli through skin with a damaged skin barrier may cause allergic sensitisation of dendritic cells from the immune system in the epidermis, resulting in an allergic response to these stimuli, in the form of asthma or atopy. Conversely, as per this hypothesis, stimulus exposure by the oral route would lead to tolerance.^17^, ^18^

There is a limited understanding of the complex interplay between the genetic and environmental factors contributing to the skin barrier hypothesis. The identification of the filaggrin gene (*FLG*) null mutations in 2006 as important risk factors for childhood AD represented a major breakthrough.^19^ *FLG* codes for pro‐filaggrin, a precursor protein which is cleaved into filaggrin, also known as filament‐aggregating protein.^20^ Filaggrin binds to keratin filament proteins in the stratum corneum, facilitating the formation of bundles and, together with the intracellular lipid layer of fatty acids, cholesterol and ceramides, is crucial in maintaining the barrier function of the skin.^13^

Loss‐of‐function mutations in *FLG* (also termed ‘null mutations’), leading to reduced levels of or absence of filaggrin, have been shown to be the strongest genetic predictor of early childhood AD, a marker of its severity, and subsequent atopy‐related disease.^21^ However, a further 236 genetic loci have been mapped to the development of AD and childhood‐onset asthma through Genome Wide Association Studies (GWAS), demonstrating the complexity of the genetic predictors of childhood atopy. Moreover, much remains unknown regarding their significance or magnitude of effect with respect to skin barrier dysfunction.^22^ A systematic review by Van Den Oord & Sheikh^23^ remains both the latest and only systematic review on skin barrier gene defects, which only investigated *FLG*, and to date, there has been no systematic review looking at skin barrier‐related genes in childhood atopic disease. A systematic review of current available evidence in this area could inform strategies for a tailored approach towards the effective management of the skin barrier, aiming to mitigate the effects of environmental exposures and reducing the burden of atopic disease in children.

### 1.1 Study aims

The aim of this systematic review and meta‐analysis was to identify the genes implicated in skin‐barrier dysfunction in childhood allergic disease, including AD, asthma, food allergy, and respiratory allergy (allergy to substances other than food), from published literature and review evidence of their effects.

## 2 MATERIALS AND METHODS

### 2.1 Search strategy

A systematic literature search was performed on 31st March 2023 using Embase, MEDLINE, Emcare, CINAHL and Cochrane Central to identify genetic association studies published between 2004 to present, investigating the role of skin barrier‐related genes in AD, asthma and allergy to food or other substances in childhood. The search strategy combined subject headings and text word synonyms for four key concepts: “child”, “gene”, “skin barrier”, and “allergy”. Language filters were applied to include papers published in English language only. The full search strategy has been included in Appendix S1.

### 2.2 Screening

Screening was conducted using systematic review software, Rayyan (Ouzzani, Hammady, Fedorowicz and Elmagarmid; Rayyan Systems; United States of America and Qatar; SCR: 017584).^24^ Initial screening of titles and abstracts was performed independently by two authors, IH and KP, and screening decisions were based on predefined inclusion and exclusion criteria, detailed below (Table 1). Full texts were obtained for abstracts that proceeded to the second stage of screening and were evaluated and screened independently by IH and KP. Screening decisions were again made on the basis of inclusion and exclusion criteria. For both stages, results were reviewed, and any disagreements were reviewed by third reviewer CH and discussed prior to making a consensus decision.

**TABLE 1:** Inclusion and Exclusion Criteria: Abstract and full text screening was performed on the basis of these criteria.

Inclusion criteria	Exclusion criteria
Population: pediatric cohort or disease group (ages 0–17 years)	Population: in‐vivo studies, animal studies
Independent variables: skin‐barrier related genetic polymorphisms or alleles	Independent variable: epigenetic measures, markers of gene or protein expression, serum biomarkers
Outcome measures: clinical manifestation of allergic disease (AD, asthma and/or wheeze, food allergy and/or respiratory allergy)	Study design: case series, narrative reviews, qualitative studies, GWAS, transcriptome‐wide association studies (TWAS)
Study design: genetic association studies (cohort or case–control studies)	Publication type: standalone conference abstracts, publication prior to 2004 (Human Genome Project)

To complete the process of full text screening, we excluded all papers related to “non‐skin barrier related genes”.

Following consultation with a team of experts in pediatrics, dermatology and genetics, we cross‐referenced genes studied in full texts against the Gene Ontology database (European Bioinformatics Institute – SCR 002811), which houses a list of genes under the term ‘skin barrier’,^25^ and only included papers relating to these named genes.

### 2.3 Quality assessment

We used The Quality of Genetic Studies (Q‐Genie) tool for quality assessment of included full texts. Q‐Genie is a validated quality assessment tool for genetic association studies, shown to reduce study heterogeneity and improve accuracy of results' estimates when applied for quality assessment in previous meta‐analyses.^26^, ^27^ Further details are supplied in Appendix S1.

### 2.4 Data extraction

Data was extracted independently by IH and KP, utilizing blinded systematic review software, Covidence (Veritas Health Innovation Ltd.; Australia – SCR: 016484), to reduce bias and likelihood of human error, details of which are supplied in Appendix S1.

### 2.5 Outcome measures

For each outcome measure: effect size, that is, odds ratio (OR) or risk ratio (RR) and measure of statistical precision/significance, that is, confidence intervals (CIs) and/or p‐values.

### 2.6 Data analysis

Data analysis consisted of meta‐analysis and narrative synthesis. Multiple meta‐analyses were conducted to investigate the overall association between skin‐barrier genetic polymorphisms and presence of AD, asthma, and allergy to food and/or respiratory allergy in childhood. Random‐effects meta‐analyses^28^ were conducted and reported pooled ORs for AD cohort studies, asthma, food allergy, respiratory allergy cohort studies, atopy (this variable combines food allergy and respiratory allergy), and AD case–control studies. For food allergy case–control studies and a subcategory of AD case–control studies, given the limited number of studies, fixed effects meta‐analyses were conducted. Quantitative assessment of inter‐study heterogeneity was calculated by the I^2^‐statistic. Statistical analyses were conducted using Stata/BE software (StataCorp LLC, United States of America, version 18.0–SCR 012763). Studies with insufficient clinical homogeneity for meta‐analysis were presented as narrative synthesis (Appendix S1).

### 2.7 Registration

This systematic review was registered on the PROSPERO database (SCR – 019061) and a full protocol was uploaded prior to study initiation (registration ID CRD42022355771).

## 3 RESULTS

### 3.1 Identification of studies

Initially, 8633 records published between 1946 and 31 March 2023 were identified by our systematic search. This was reduced to 6018 records after de‐duplication. 5060 studies were excluded at title‐abstract screening, resulting in 958 records proceeding to full‐text screening (Figure S1).

Following full‐text screening and subsequent quality assessment, we identified 63 eligible studies. Characteristics of included studies^19^, ^29^, ^30^, ^31^, ^32^, ^33^, ^34^, ^35^, ^36^, ^37^, ^38^, ^39^, ^40^, ^41^, ^42^, ^43^, ^44^, ^45^, ^46^, ^47^, ^48^, ^49^, ^50^, ^51^, ^52^, ^53^, ^54^, ^55^, ^56^, ^57^, ^58^, ^59^, ^60^, ^61^, ^62^, ^63^, ^64^, ^65^, ^66^, ^67^, ^68^, ^69^, ^70^, ^71^, ^72^, ^73^, ^74^, ^75^, ^76^, ^77^, ^78^, ^79^, ^80^, ^81^, ^82^, ^83^, ^84^, ^85^, ^86^, ^87^, ^88^, ^89^ and their quality assessment outcomes are presented in Table S2. Independent variables were polymorphisms and/or LOF mutations in *FLG, FLG‐2*, or interleukin‐18 (*IL18*).

This review is reported in line with Preferred Reporting Items for Systematic Reviews and Meta‐analyses (PRISMA) guidelines (Figure S1).

### 3.2 Meta‐analysis

Twenty‐nine studies were eligible for meta‐analysis to investigate the effect of skin‐barrier related genes on childhood AD, asthma, food allergy and respiratory allergy. Due to the range of study types and outcome measures, multiple meta‐analyses were conducted. All *FLG* polymorphisms were combined in our analysis. A random effects meta‐analysis was performed due to significant study heterogeneity, including study design.

### 3.3 *FLG* and AD

Random effects meta‐analysis was conducted on the statistical interaction between *FLG* null mutations and AD. Analyses were conducted separately for cohort studies and case–control studies, as odds ratios and risk ratios are not directly comparable.

**FIGURE 1:**
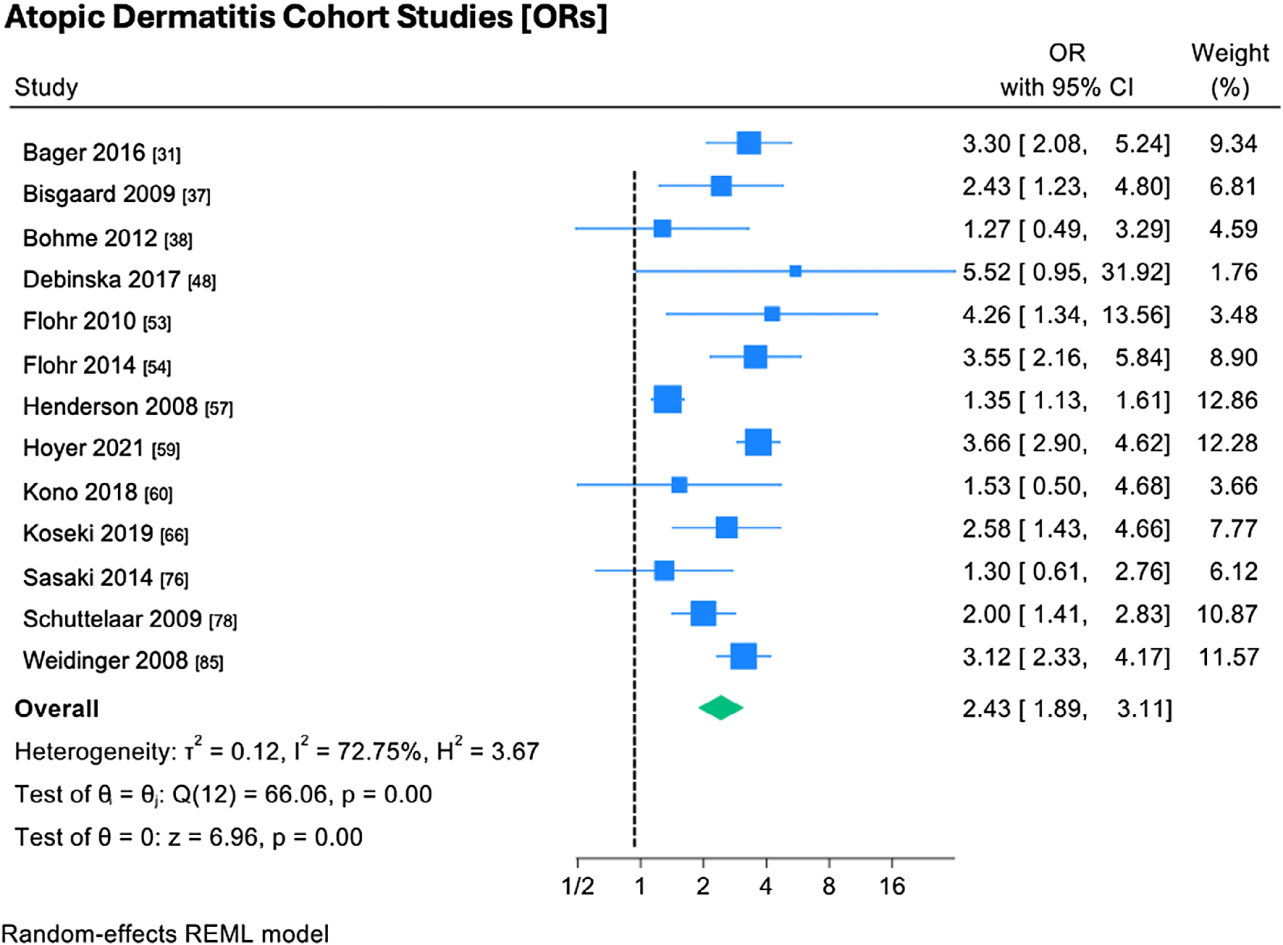
The association between *FLG* mutations and atopic dermatitis (cohort studies). This forest plot demonstrates random‐effects meta‐analysis on the association between *FLG* mutations and atopic dermatitis in cohort studies, showing individual and pooled effect size, with corresponding confidence intervals. Testing (test of θ = 0) was performed to determine if the pooled effect size was indicative of true effect size. Results of testing for overall heterogeneity and test of θ_i_ (homogeneity of specific study effect sizes) additionally provided. (OR, odds ratio; 95% CI, confidence interval).

Thirteen AD cohort studies were meta‐analyzed,^31^, ^37^, ^38^, ^48^, ^53^, ^54^, ^57^, ^60^, ^65^, ^66^, ^76^, ^78^, ^85^ with ten showing associations between *FLG* null mutations and AD which were statistically significant (Figure 1). The pooled effect size was large (OR 2.42, 95% CI 1.89–3.11), with significant study heterogeneity (*I* ^2^ = 72.75%, *p* = <.05).

Separate meta‐analyses were performed on AD case–control studies and two further AD cohort studies utilizing alternative outcome measures (risk ratios). Results are supplied in Appendix S1.

### 3.4 *FLG* and asthma/wheeze

**FIGURE 2:**
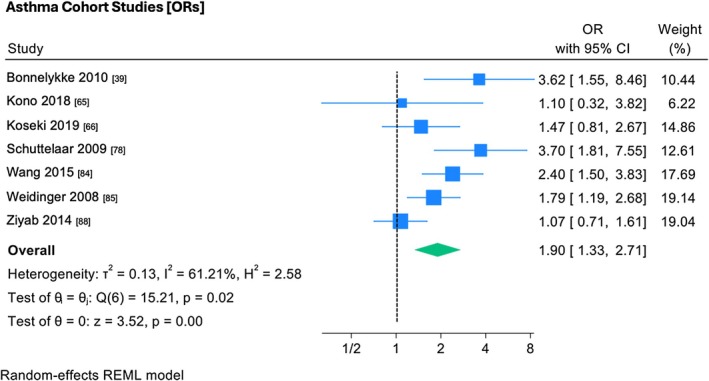
The association between *FLG* mutations and asthma/wheeze (cohort studies). This forest plot demonstrates random‐effects meta‐analysis on the association between *FLG* mutations and asthma/wheeze in cohort studies, showing individual and pooled effect size, with corresponding confidence intervals. Testing (test of θ = 0) was performed to determine if the pooled effect size was indicative of true effect size. Results of testing for overall heterogeneity and test of θ_i_ (homogeneity of specific study effect sizes) additionally provided. (OR, odds ratio; 95% CI, confidence interval).

Random effects meta‐analysis was conducted on the interaction between *FLG* polymorphisms and asthma and/or wheeze. All seven included studies^39^, ^65^, ^66^, ^78^, ^84^, ^85^, ^88^ were cohort studies. Five out of seven cohort studies showed a statistically significant association between *FLG* null mutations and asthma (Figure 2). The pooled effect size (OR 1.90, 95% CI 1.33–2.71) was statistically significant. There was notable study heterogeneity (*I* ^2^ = 61.21%, *p* = .02).

### 3.5 *FLG* and atopy

Random effects meta‐analysis was conducted on the interaction between *FLG* polymorphisms and atopy, where an effect size combining food allergy and respiratory allergy was provided. Combined data for food and respiratory allergy was only used where disaggregated data was unavailable.

**FIGURE 3:**
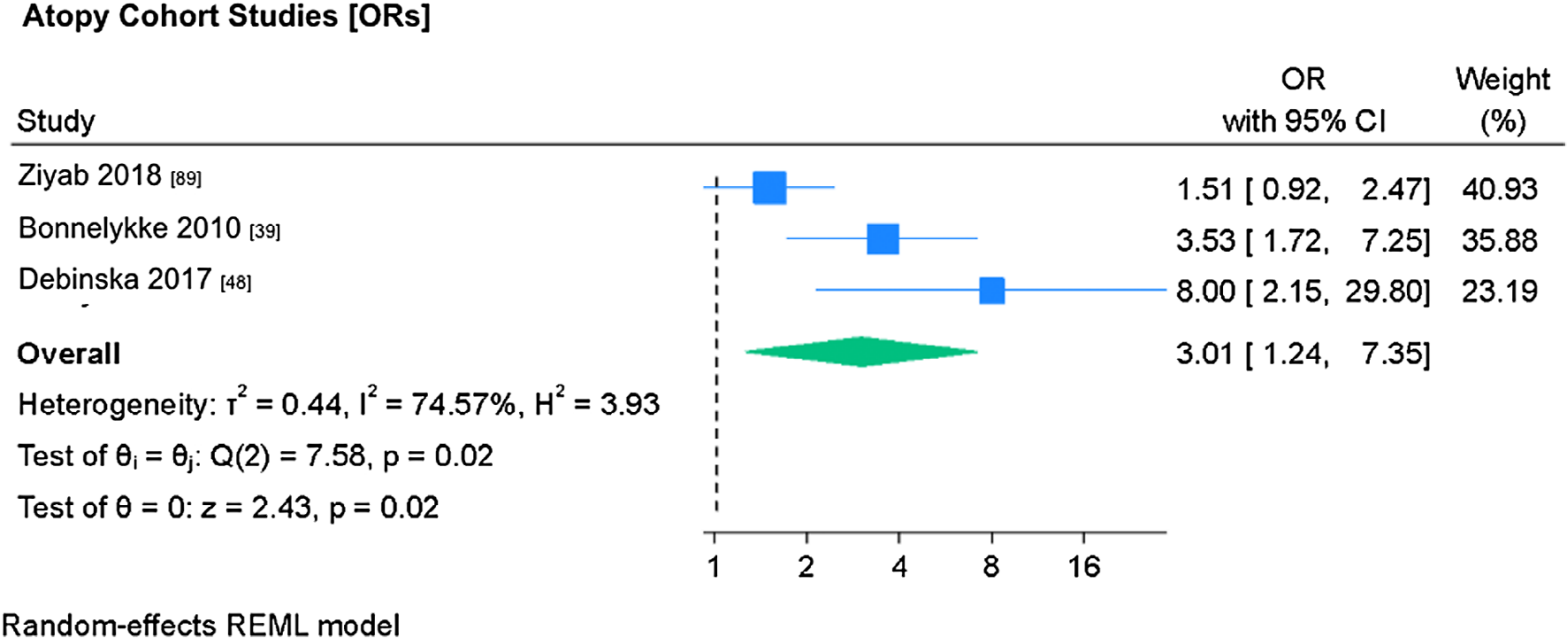
The association between *FLG* mutations and atopy (cohort studies). This forest plot demonstrates random‐effects meta‐analysis on the association between *FLG* mutations and atopy in cohort studies, showing individual and pooled effect size, with corresponding confidence intervals. Testing (test of θ = 0) was performed to determine if the pooled effect size was indicative of true effect size. Results of testing for overall heterogeneity and test of θ_i_ (homogeneity of specific study effect sizes) additionally provided. (OR, odds ratio; 95% CI, confidence interval).

All three analyzed studies^39^, ^49^, ^89^ were cohort studies. Bonnelykke^39^ (OR 3.53, 95% CI 1.72–7.25) and Debinska^48^ (OR 2.15, 95% CI 215–29.8) demonstrated a significant association between *FLG* null mutations and combined food and respiratory allergies (Figure 3). Meta‐analysis demonstrated a large pooled effect size (OR 3.01, 95% CI 1.24–7.35) which was statistically significant, and substantial heterogeneity (*I* ^2^ = 74.57%, *p* = .02).

### 3.6 *FLG* and food allergy

Random‐effects meta‐analysis was conducted on the interaction between *FLG* null mutations and food allergy. Separate analyses were conducted on cohort studies and case–control studies due to differences in study design.

**FIGURE 4:**
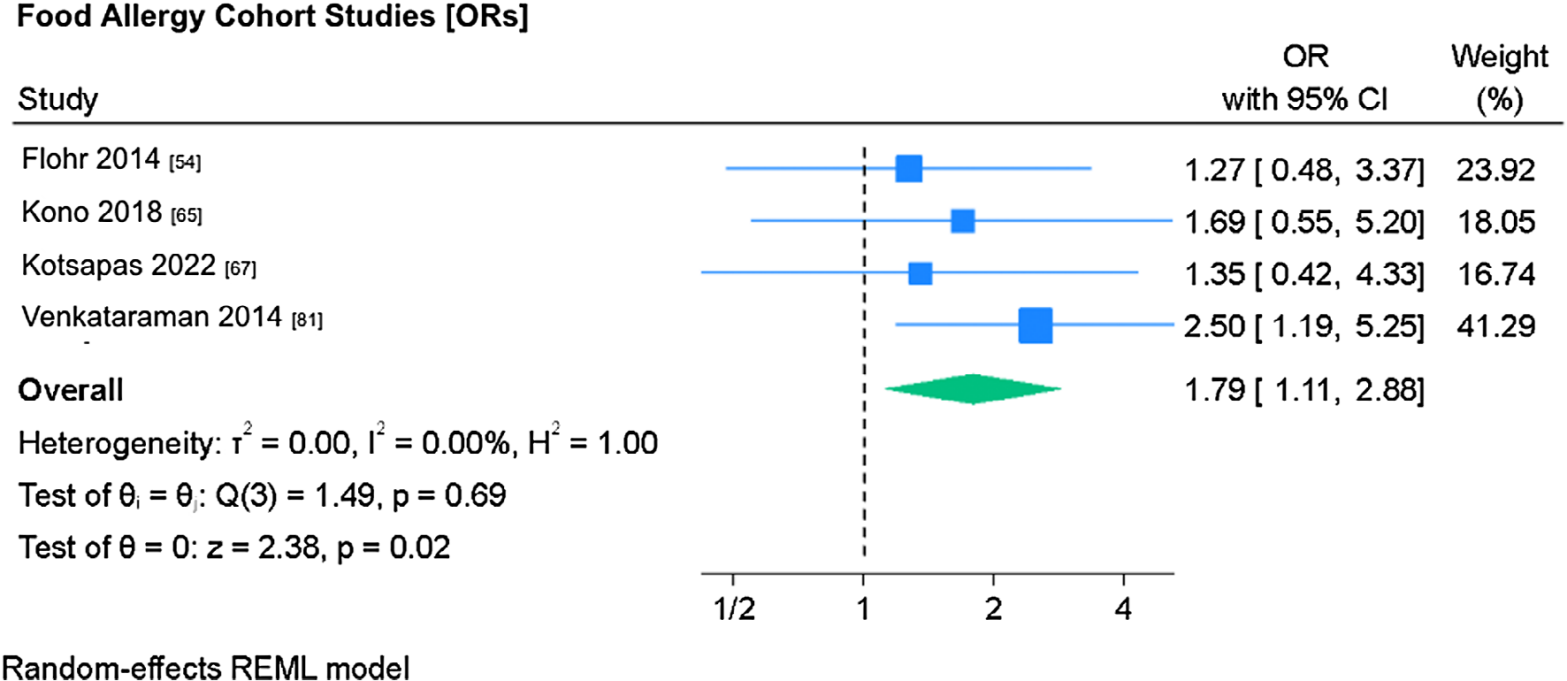
The association between *FLG* mutations and food allergy (cohort studies). This forest plot demonstrates random‐effects meta‐analysis on the association between *FLG* mutations and food allergy in cohort studies, showing individual and pooled effect size, with corresponding confidence intervals. Testing (test of θ = 0) was performed to determine if the pooled effect size was indicative of true effect size. Results of testing for overall heterogeneity and test of θ_i_ (homogeneity of specific study effect sizes) additionally provided. (OR = odds ratio; 95% CI = confidence interval).

There were four food allergy cohort studies meta‐analyzed.^54^, ^65^, ^67^, ^81^ Three of four cohort studies showed no statistically significant association, with only Venkataraman^81^ showing statistical significance (OR 2.50, 95% CI 1.19–2.88) (Figure 4). Analysis showed an association between food allergy and *FLG* null mutations, which was statistically significant at the 5% level [OR 1.79, (95% CI 1.11–2.88)] between food allergy and *FLG* null mutations. Meta‐analysis demonstrated no study heterogeneity (*I* ^2^ = 0%) (*p* = .65).

**FIGURE 5:**
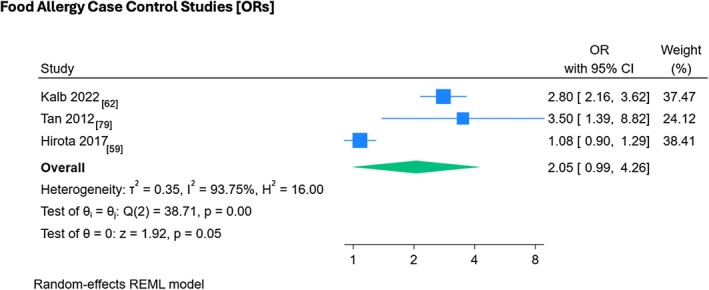
The association between *FLG* mutations and food allergy (case–control studies). This forest plot demonstrates random effects meta‐analysis on the association between *FLG* mutations and food allergy in case–control studies. This forest plot demonstrates individual and pooled effect size, with corresponding confidence intervals. Testing (test of θ = 0) was performed to determine if the pooled effect size was indicative of true effect size. Test of θ_i_ (homogeneity of specific study effect sizes) additionally provided. (RR, risk ratio; 95% CI, confidence interval).

Three food allergy case–control studies were meta‐analyzed by random effects meta‐analysis,^59^, ^62^, ^79^ with two studies demonstrating significant associations between food allergy and *FLG* null mutations (OR 2.80, 95% CI 2.16–3.62) and (OR 3.50, 95% CI 1.39–8.82) individually (Figure 5).^62^, ^79^ The pooled effect size did not show statistical significance, (OR 2.05, 95% CI 0.99–4.26) and there was significant heterogeneity (*I* ^2^ = 93.75%, *p* = .00).

### 3.7 FLG and respiratory allergy

**FIGURE 6:**
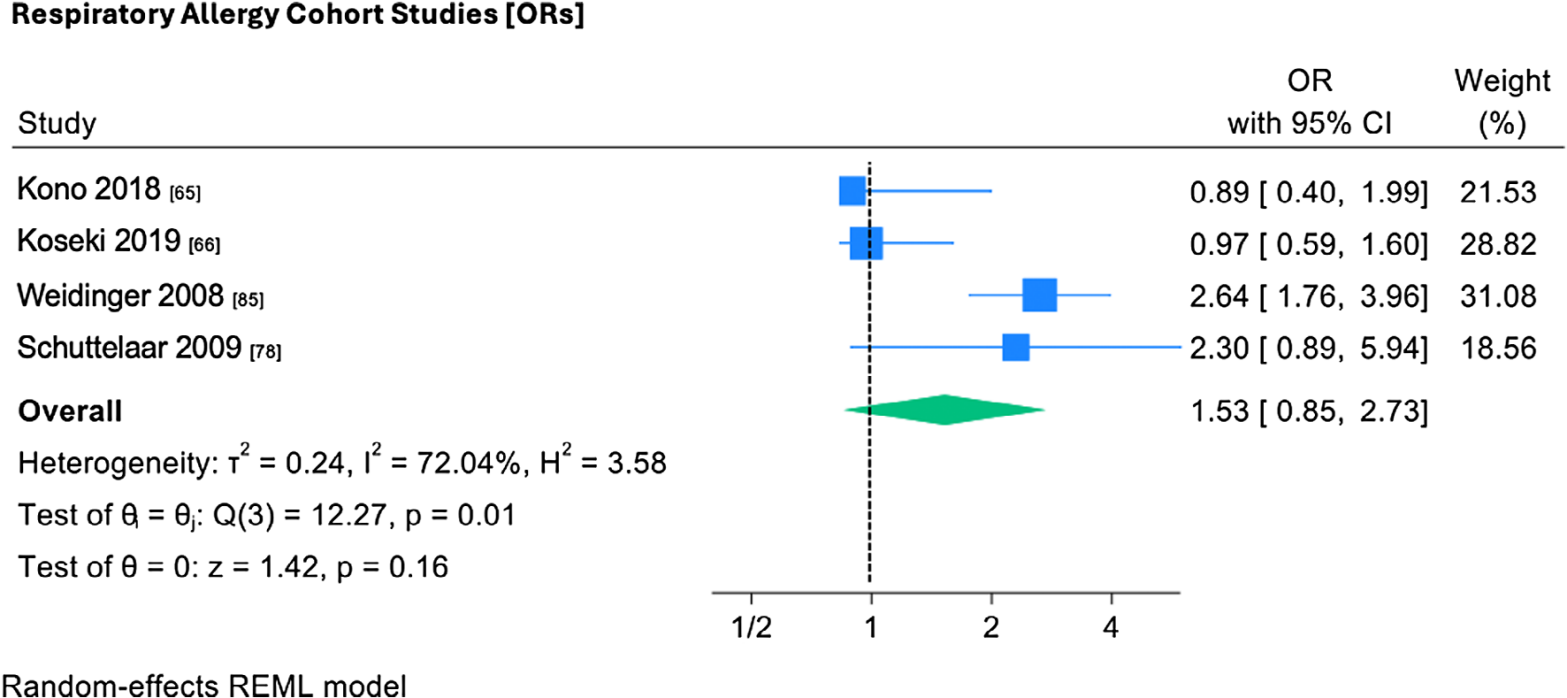
The association between *FLG* mutations and respiratory allergy (cohort studies). This forest plot demonstrates random‐effects meta‐analysis on the association between *FLG* mutations and respiratory allergy cohort studies, showing individual and pooled effect size, with corresponding confidence intervals. Testing (test of θ = 0) was performed to determine if the pooled effect size was indicative of true effect size. Results of testing for overall heterogeneity and test of θ_i_ (homogeneity of specific study effect sizes) additionally provided. (OR, odds ratio; 95% CI, confidence interval).

Random effects meta‐analysis was conducted on the interaction between *FLG* polymorphisms and respiratory allergy (Figure 6).^65^, ^66^, ^78^, ^85^ All four analyzed studies were cohort studies in which respiratory allergy outcome measures (environmental allergens) were analyzed. Three of four analyzed studies^65^, ^66^, ^81^ showed no statistically significant association. Weideinger^85^ was the only study to demonstrate a relatively large effect size (OR 2.64, 95% CI 1.76–3.96). The pooled odds ratio (OR 1.53, 95% CI 0.85–2.73) demonstrated no statistically significant overall association between *FLG* null mutations and respiratory allergy. Additionally, heterogeneity analysis (*I* ^2^ = 72.04%, *p* = .01) demonstrated substantial heterogeneity among studies, which was statistically significant.

### 3.8 Narrative synthesis

Thirty studies were suitable for inclusion in this review but were not included in the meta‐analysis due to differing outcome measures and statistical analyses. These studies were included in our narrative synthesis. All included studies investigated the impact of *FLG*, *FLG‐2*, or *IL18* on childhood AD, wheeze or asthma, and/or allergy. Our search did not find studies investigating any other skin‐barrier related genes meeting our inclusion criteria. Full narrative synthesis can be found in Appendix S1.

## 4 DISCUSSION

This is the first systematic review and meta‐analysis of skin barrier associated genes implicated in childhood atopic disease. There is established evidence for *FLG* in AD, asthma and food allergy in childhood. Our review, to include both meta‐analysis and narrative synthesis, found mixed results for *FLG* in respiratory allergy; however, this may have been influenced by the small number of studies and heterogeneity between studies. Other genes identified by our search were *FLG‐*2 and *IL‐18*, of which one study each met inclusion criteria for narrative synthesis. Currently, further evidence is required to appreciate the role of *FLG‐2* and *IL‐18* in childhood atopy. We did not identify evidence for other skin barrier‐related gene variation in childhood atopy.

### 4.1 *FLG* polymorphisms and presence of AD

Our analysis highlighted a significant association between *FLG* mutations and the presence of AD in childhood.

There is considerable variation between included studies, reflecting differences in study design, cohort size and the number and type of *FLG* null mutations analyzed, with noticeable global variation. The number of mutations genotyped ranged from three to twenty‐two, and the most common mutations varied between populations. For example, Turkish studies did not detect the R501X mutation, which is one of the most prevalent *FLG* null mutations in European populations.^80^ The considerable heterogeneity of our meta‐analysis emphasizes the importance of considering mutation panels, and population diversity when interpreting results, emphasizing the need for genetic studies with large sample sizes and diverse cohorts.

Our results indicated early onset AD was linked to *FLG* null mutations in several cohorts, including a 2022 Singaporean study by Cai et al.^44^ In contrast, the American PEER study,^72^ which genotyped children with early onset AD for any of the four most common *FLG* null mutations in European populations (R501X, 2282del4, R2447X, and S3247X), demonstrated an increased likelihood of early onset AD in children with one *FLG* null mutation (heterozygotes) (OR 1.74, 95% CI 1.02–2.96). Those with two null mutations (homozygotes or compound heterozygotes) were found to have a three‐fold increase in early onset AD risk, although this was not statistically significant (OR 3.03, 95% CI 0.70–13.16).^81^ There were inconsistencies in study definitions of “early‐onset”, which ranged between <3 months and ≤2 years old, reflecting the need for standardized definitions and large sample sizes. In addition, the criteria used to diagnose AD varied between studies, ranging from clinician judgment (‘physician‐diagnosed’), UK Working Party and Hanifin and Rajka criteria. This implies a possible variation in diagnostic threshold for AD between studies, and is likely to contribute towards study heterogeneity. For instance, it has been noted that the UKWP criteria are more restrictive than other diagnostic criteria^90^ and may therefore exclude non‐classical phenotypes of AD.

Whilst several included studies correlated the presence of *FLG* mutations with more severe AD,^30^, ^34^, ^50^, ^68^ others showed no statistically significant correlation.^36^ In some studies, AD severity was largely evaluated by validated scoring systems^88^ such as SCORAD (SCORing Atopic Dermatitis)^28^, ^32^, ^43^, ^47^, ^78^, ^83^ and EASI (Eczema Area and Severity Index),^68^ whilst other studies used locally derived methods of assessing AD severity,^33^, ^41^, ^44^, ^72^, ^75^ such as parent‐reported surveys or symptom‐free periods, making comparisons between severities more subjective.

The association between *FLG* mutations and AD persistence was more consistent. The presence of four *FLG* null mutations was linked to persistent AD in the PEER study, with persistence defined as the absence of symptom‐free periods.^72^ These findings suggest that *FLG* mutations may play a role in the chronic progression of AD.

Our findings of association between *FLG* and AD are in keeping with other systematic reviews investigating genes associated with AD in child and adult populations.^91^, ^92^ With respect to additional skin‐barrier‐associated genes, Pontikas et al. report consistent associations between AD and *FLG‐2*,^91^ which was found in only one included paper for our review and thus could not be meta‐analyzed, *CLDN‐1* as a result of a whole‐exome sequencing (WES) study,^93^ and both *CLDN‐1* and *CLDN‐4* in a genetic association study with the majority of adult participants.^91^ Of note, these reviews additionally report associations between AD and genes not recognized to be skin‐barrier‐associated.^91^, ^92^

### 4.2 Asthma and wheeze

Meta‐analysis of *FLG* polymorphisms in relation to asthma and/or wheeze demonstrated a significant pooled effect size, but with significant study heterogeneity, which may reflect wide variations in studied populations. This may also reflect the pooling of the associations of a wide range of *FLG* mutations, specifically R501X, 2282del4, R2447X, P478S, S3247X, Arg501X, 3321delA, 3702delG, Ser1695X, Gln1701X, Ser1695X, Ser2554X, Ser2889X, Ser3296X, Lys4022X, Q1790X, 441‐442delA, Gln1790X, and Lys4022X.^19^, ^38^, ^62^, ^63^, ^79^, ^80^, ^83^

Narrative synthesis highlighted mixed results for *FLG*, which may be explained by the differences in population ethnicities, sample sizes and, similarly to meta‐analysis, differences in specific *FLG* mutations investigated between studies. Additionally, epigenetic changes have been associated with childhood asthma with current evidence proposing a role for environmental factors, such as air pollutants, passive smoking and pet ownership, in gene methylation.^95^, ^96^ Although not studied in this review, this would be a valuable area of focus for future work looking at skin‐barrier epigenetics in asthma.

Whilst our review focused on candidate gene studies, systematic reviews of GWAS comprise the mainstay of pre‐existing literature in asthma and/or wheeze genetics. In a U.K.‐based meta‐analysis of GWAS, Granell et al. highlight 85 SNPs to be associated with four different phenotypes of preschool wheeze with a novel locus close to ANXA‐1,^97^ however, for all SNPs, their adjacent genes are not skin‐barrier associated, as per the aforementioned Gene Ontology database. Additionally, it should be noted that *FLG* is not adjacent to the SNPs detected here, despite this meta‐GWAS including a large number of participants with exclusive European ancestry.

A meta‐analysis of GWAS spanning non‐European populations found 169 significant loci to be associated with asthma, of which 2 are related to the skin barrier: *HRNR* and *CYP26B1*.^98^ It should, however, be noted that this meta‐analysis included GWAS rather than candidate gene studies as in our review. Moreover, study populations were composed of adults and children; therefore, this meta‐analysis was not specific to childhood asthma.

### 4.3 Food Allergy

Meta‐analysis of cohort studies revealed a significant association between food allergy and the presence of *FLG* null mutations. This was not the case in the meta‐analysis of case–control studies. The mixed results observed from the five studies included in our narrative synthesis may have been due to differences in mutation frequencies and allergen exposure.^40^, ^59^, ^61^, ^74^, ^80^ Among children with coexistent AD, *FLG* null mutations were correlated with severe allergic reactions, such as anaphylaxis, in Italian cohorts, suggesting a potential role in severe food allergy phenotypes.^29^

Our results were consistent with skin‐barrier related genes detected in existing literature; a systematic review encompassing 32 pediatric studies (a combination of candidate gene studies and GWAS) found that polymorphisms in the *FLG* gene were associated with food allergy. Despite multiple other genes and gene areas of interest, only *FLG* was associated with skin‐barrier function.^99^

Discussion of respiratory allergy and atopy is presented in Appendix S1.

## 5 STRENGTHS AND LIMITATIONS

This is the first systematic review after 2009 to explore the relationship between *FLG* mutations and childhood atopy.

However, we may have excluded genes with functions relating to the skin barrier which are not yet recognized by the Gene Ontology Skin Barrier Database, which was utilized as an internationally‐validated genetic database. The sample sizes of some subgroups were relatively small, which may impact the internal validity of our findings.

In total, 49 studies were excluded through language filters. However, from screening abstracts, which were written in English, none of these studies otherwise met our inclusion criteria, largely due to study design, genes studied not in the skin‐barrier database utilized or that there was only an abstract available with no corresponding full text. We therefore conclude that no major non‐English studies of relevance were omitted due to this approach.

Additionally, while most studies genotyped between three and ten *FLG* loss‐of‐function mutations, only one study analyzed 22 *FLG* polymorphisms, which suggests that rarer but potentially clinically relevant variants may not have been reported by the other studies. Significant global variation was noted with respect to the most common FLG mutations studied, which reflects diversity in genotypes between populations, and also may contribute towards study heterogeneity. We recognize that the study heterogeneity observed in meta‐analyses presents challenges in meta‐analysis. Due to the small number of papers in each meta‐analysis, subgroup analyses and stratified pooled estimates were not possible.

**Table:** 

**Research agenda for future studies** Standardizing definition and categorization of genes: this could lead to formation of mutation panels for atopic disease and, with development of personalized medicine, this categorization may have implications for future therapeutic strategies; however, this should be globally standardized. Candidate gene association studies on skin barrier‐related genes beyond *FLG*: i.e. genotyping cohorts for skin‐barrier related genes as per existing evidence and databases. Standardization of methodology: utilizing the same diagnostic and severity assessment tools would reduce heterogeneity between studies, hence allowing for more rigorous comparison of data. Representation of global populations in research due to variation in genotypes between different ethnicities, across all atopic diseases, that is, AD, asthma, food allergy, respiratory allergy.

We also acknowledge that combining *FLG* polymorphisms and mutations for analysis, rather than studying individual effects, may have influenced results. Whilst a random effects model of meta‐analysis accounted for variations in sample sizes and study design, it emphasized the need for consistent study designs with large sample sizes to increase statistical power for detecting smaller gene effects.

## 6 CONCLUSIONS

In conclusion, our work demonstrates a lack of sufficient high‐quality research on non‐FLG skin barrier‐related genes, representing an important area for further work. This systematic review and meta‐analysis highlights several areas of future research for prioritization, such as standardizing definition and categorization of genes, candidate gene association studies on skin barrier‐related genes, standardization across study design, diverse global populations with varying genotypes and varying classifications of atopic disease. In particular, global variability in common *FLG* mutations has implications for population‐specific risk stratification and personalized medicine beyond predominantly Eurocentric treatment strategies. This systematic review synthesizes evidence implicating *FLG* mutations in the pathogenesis of childhood atopic disease. Although significant progress has been made in understanding the role of *FLG* variation in children's atopy, further investigation is essential to define the role of additional skin barrier‐associated genes.

An intact skin barrier protects against the entry of environmental allergens. A skin barrier defect from birth is likely to affect the body's response to environmental allergens, and a study of this response in those with a skin barrier defect at birth may help us better understand the process of allergic sensitisation in early life and guide the development of personalised medicine for this cohort of patients.

## AUTHOR CONTRIBUTIONS

**I. Husain:** Conceptualization; investigation; writing – original draft; methodology; validation; writing – review and editing; visualization; software; formal analysis; project administration; data curation; resources. **K. Patel:** Conceptualization; investigation; writing – original draft; writing – review and editing; visualization; validation; methodology; software; formal analysis; project administration; data curation; resources. **C. Holden:** Conceptualization; investigation; writing – review and editing; validation; visualization; methodology; supervision. **L. Jervis:** Conceptualization; investigation; writing – original draft; methodology; validation; visualization; writing – review and editing; software; formal analysis; project administration; data curation; resources. **K. Barnard:** Methodology; validation; software; formal analysis; project administration; resources; data curation. **E. Youssef:** Supervision; resources; formal analysis; methodology. **J. Felton:** Conceptualization; investigation; methodology; validation; writing – review and editing; supervision; resources. **S. Bremner:** Writing – review and editing; methodology; validation; conceptualization; investigation; software; formal analysis; supervision; data curation; resources. **S. J. Brown:** Conceptualization; investigation; methodology; validation; visualization; writing – review and editing; software; formal analysis; data curation; supervision; resources. **S. Mukhopadhyay:** Conceptualization; investigation; validation; visualization; methodology; writing – review and editing; software; formal analysis; project administration; supervision; resources; data curation; writing – original draft.

## FUNDING INFORMATION

No specific funding was received for this work. This publication is the work of the authors and all authors will serve as guarantors for the contents of this paper.

## CONFLICT OF INTEREST STATEMENT

The authors declare no conflict of interest in relation to this work.

## Supporting information


Appendix S1.
