# Supplementary material for: Skin barrier‐related genes in childhood atopic dermatitis, asthma, and allergy: A systematic review and meta‐analysis
Source: Pediatr Allergy Immunol. 2026 Apr 23;37(4):e70326. doi: 10.1111/pai.70326 (PMC13105850; doi:10.1111/pai.70326)
Supplement: Supplementary file 1 — Appendix S1. [file PAI-37-e70326-s001.docx]

**Supplementary Material**

**Supplementary Methods**

**Search strategies**

*Note that field 14 refers to the skin-barrier related candidate genes from existing GWAS.*

**Embase via Ovid**

**31.03.2023**

| 1 | exp child/ or exp juvenile/ or exp adolescent/ | 3994816 |
| --- | --- | --- |
| 2 | exp newborn/ or exp infant/ | 1125996 |
| 3 | (infant* or newborn or neonat* or neo-nat* or baby or babies or child* or toddler* or pre?school* or pediatri* or paediatri* or adolescen* or teenage* or juvenile*).mp. | 4988100 |
| 4 | 1 or 2 or 3 | 5052417 |
| 5 | exp gene/ or "genetic association"/ or exp "human genetics"/ or "genetic variability"/ or "genetic susceptibility"/ or "genetic marker"/ or "genetic analysis"/ or exp DNA/ | 2598328 |
| 6 | (gene* or genome* or exome* or exon or Transcriptome* or loci or "Single Nucleotide Polymorphism" or "Deoxyribonucleic acid" or DNA or Chromosom*).mp. | 10345511 |
| 7 | 5 or 6 | 10371134 |
| 8 | ((skin or epidermal) adj4 barrier*).mp. | 15384 |
| 9 | ("transepidermal water loss" or TEWL).mp. | 5469 |
| 10 | "Stratum corneum hydration".mp. | 581 |
| 11 | "skin permeability"/ or "skin barrier dysfunction"/ or "skin allergy"/ or "skin sensitization"/ | 35887 |
| 12 | 8 or 9 or 10 or 11 | 51894 |
| 13 | 7 and 12 | 12485 |
| 14 | (ACTL9 or ADO or AFF1 or AIMP1P2 or ALDH7A1P4 or ANKRD11P1 or AOAH or AP5B1 or ATP6V1G2-DDX39B or BCLAF1P1 or BHLHE40 or C3orf35 or CAPSL or CARD11 or CAVIN1 or CCDC80 or CCR7 or CD207 or CD247 or CDH13 or CHCHD3P1 or CHROMR or CHST8 or CIART or CLEC16A or CPE or CRCT1 or CREB5 or CSF2RB or CSGALNACT1 or CTIF or CTNNA2 or CYP24A1 or D2HGDH or DCHS2 or DDX39B or DMRTA1 or EEF1A1P27 or EFHC1 or EGR2 or EHMT1 or EMSY or ERBB3 or ERG or ETS1 or EVI5 or FAM177A1 or FGF20 or *FLG* or *FLG*-AS1 or FOXCUT or GAPDHP41 or GLB1 or GOLGA4 or GPHN or GPSM3 or GRM4 or HLA-B or HLA-DQA1 or HLA-DRB1 or HMSD or IKZF3 or IL13 or IL15RA or *IL18*R1 or *IL18*RAP or IL1R1 or IL1RL1 or IL21 or IL22 or IL2RA or IL6R or IL7R or INPP5D or IQGAP1 or IRAK1BP1 or ITGB8 or KCND2 or KCTD8 or KIAA1109 or KIF3A or KPNA3 or KRT8P26 or KRT8P37 or LARP7P4 or LAYN or LCE1E or LCE1F or LCE3A or LCE3E or LCE5A or LCEP4 or LGMNP1 or LINC00299 or LINC00603 or LINC00624 or LINC00709 or LINC00824 or LINC00861 or LINC01080 or LINC01143 or LINC01503 or LINC01653 or LINC01882 or LINC01993 or LINC02042 or LINC02055 or LINC02098 or LINC02571 or LINC02676 or LINC02757 or LRRC32 or MDM1 or MEI4 or MFN2 or MGMT or MICB or MIR302F or MIR4435-2HG or MIR5708 or MRPS21 or MSL3P1 or MTND5P15 or MYT1L or NAA35 or NBAS or NBPF17P or NCF4 or NCF4-AS1 or NCKAP5 or NFILZ or NLRP10 or NONOP2 or OR10A3 or 2W5P or OVOL1 or PA2G4 or PBX2 or PFDN4 or PGLYRP4 or PHB or PHBP7 or PON1 or PPP2R3C or PRKRA or PRORP or PRR5L or PTPN2 or PTPRN2 or PUDPP2 or PUS10 or R3HCC1L or RAD50 or RASIP1 or RBBP4P4 or RBMS3 or RCBTB1 or REL or RN7SL335P or RN7SL352P or RN7SL474P or RNA5SP144 or RNF111 or RNU1-141P or RNU6-1213P or RNU6-408P or RPL21P17 or RPL23AP39 or RPL41 or RPS3AP21 or RSL24D1 or RTEL1 or RTEL1-TNFRSF6B or S100A11 or SCAPER or SDR42E1P5 or SEMA6C or SERPINB10 or SFRP2 or SIK2 or SLC25A38P1 or SLC25A46 or SLC9A4 or SMAD3 or SMAD7 or SMARCA4 or SMARCE1 or SOCAR or SOCS3 or SPRR1B or SPRR3 or SPRY2 or STAT3 or STAT6 or STEAP2-AS1 or TESPA1 or TGM3 or TH2LCRR or THEMIS or TLR1 or TNFRSF11A or TNFRSF6B or TNXB or TRAF3 or TRIB1 or TRPM8 or TSBP1 or TSBP1-AS1 or WDR36 or XIRP2 or XIRP2-AS1 or Y_RNA or ZBTB38 or ZGLP1 or ZNF217 or ZNF365 or ZNF652 or OLFM1 or FCN1 or CDSN or CTLA-4 or DOCK8 or DSG1 or DSP or FOXP3 or IFNGR1 or IL4RA or IVL or KRT10 or MALT1 or PGM3 or RAG1 or RAG2 or SPINK5 or TARC or TSLP).mp. | 319354 |
| 15 | 13 or 14 | 330284 |
| 16 | exp "atopic dermatitis"/ or exp eczema/ or exp asthma/ or exp allergic asthma/ or exp atopy/ or exp food allergy/ or exp skin allergy/ or allergy/ or exp wheezing/ | 489948 |
| 17 | ("atopic* dermatitis" or asthma* or atopy or allerg* or wheez* or eczema).mp. | 706780 |
| 18 | 16 or 17 | 709450 |
| 19 | 4 and 15 and 18 | 5018 |

**Medline via Ovid**

**31.03.2023**

| 1 | exp child/ or adolescent/ | 3342021 |
| --- | --- | --- |
| 2 | exp infant/ | 1244097 |
| 3 | (infant* or newborn or neonat* or neo-nat* or baby or babies or child* or toddler* or pre?school* or pediatri* or paediatri* or adolescen* or teenage* or juvenile*).mp. | 4816165 |
| 4 | 1 or 2 or 3 | 4816165 |
| 5 | exp Genes/ or exp Genetic Predisposition to Disease/ or exp Genetics/ or exp Genetic Variation/ or Genetic Markers/ or exp DNA/ | 2496419 |
| 6 | (gene* or genome* or exome* or exon or Transcriptome* or loci or "Single Nucleotide Polymorphism" or "Deoxyribonucleic acid" or DNA or Chromosom*).mp. | 8476587 |
| 7 | 5 or 6 | 8555295 |
| 8 | ((skin or epidermal) adj4 barrier*).mp. | 9680 |
| 9 | ("transepidermal water loss" or TEWL).mp. | 3515 |
| 10 | "Stratum corneum hydration".mp. | 400 |
| 11 | Skin Absorption/ | 12657 |
| 12 | 8 or 9 or 10 or 11 | 23100 |
| 13 | 7 and 12 | 5027 |
| 14 | (ACTL9 or ADO or AFF1 or AIMP1P2 or ALDH7A1P4 or ANKRD11P1 or AOAH or AP5B1 or ATP6V1G2-DDX39B or BCLAF1P1 or BHLHE40 or C3orf35 or CAPSL or CARD11 or CAVIN1 or CCDC80 or CCR7 or CD207 or CD247 or CDH13 or CHCHD3P1 or CHROMR or CHST8 or CIART or CLEC16A or CPE or CRCT1 or CREB5 or CSF2RB or CSGALNACT1 or CTIF or CTNNA2 or CYP24A1 or D2HGDH or DCHS2 or DDX39B or DMRTA1 or EEF1A1P27 or EFHC1 or EGR2 or EHMT1 or EMSY or ERBB3 or ERG or ETS1 or EVI5 or FAM177A1 or FGF20 or *FLG* or *FLG*-AS1 or FOXCUT or GAPDHP41 or GLB1 or GOLGA4 or GPHN or GPSM3 or GRM4 or HLA-B or HLA-DQA1 or HLA-DRB1 or HMSD or IKZF3 or IL13 or IL15RA or *IL18*R1 or *IL18*RAP or IL1R1 or IL1RL1 or IL21 or IL22 or IL2RA or IL6R or IL7R or INPP5D or IQGAP1 or IRAK1BP1 or ITGB8 or KCND2 or KCTD8 or KIAA1109 or KIF3A or KPNA3 or KRT8P26 or KRT8P37 or LARP7P4 or LAYN or LCE1E or LCE1F or LCE3A or LCE3E or LCE5A or LCEP4 or LGMNP1 or LINC00299 or LINC00603 or LINC00624 or LINC00709 or LINC00824 or LINC00861 or LINC01080 or LINC01143 or LINC01503 or LINC01653 or LINC01882 or LINC01993 or LINC02042 or LINC02055 or LINC02098 or LINC02571 or LINC02676 or LINC02757 or LRRC32 or MDM1 or MEI4 or MFN2 or MGMT or MICB or MIR302F or MIR4435-2HG or MIR5708 or MRPS21 or MSL3P1 or MTND5P15 or MYT1L or NAA35 or NBAS or NBPF17P or NCF4 or NCF4-AS1 or NCKAP5 or NFILZ or NLRP10 or NONOP2 or OR10A3 or 2W5P or OVOL1 or PA2G4 or PBX2 or PFDN4 or PGLYRP4 or PHB or PHBP7 or PON1 or PPP2R3C or PRKRA or PRORP or PRR5L or PTPN2 or PTPRN2 or PUDPP2 or PUS10 or R3HCC1L or RAD50 or RASIP1 or RBBP4P4 or RBMS3 or RCBTB1 or REL or RN7SL335P or RN7SL352P or RN7SL474P or RNA5SP144 or RNF111 or RNU1-141P or RNU6-1213P or RNU6-408P or RPL21P17 or RPL23AP39 or RPL41 or RPS3AP21 or RSL24D1 or RTEL1 or RTEL1-TNFRSF6B or S100A11 or SCAPER or SDR42E1P5 or SEMA6C or SERPINB10 or SFRP2 or SIK2 or SLC25A38P1 or SLC25A46 or SLC9A4 or SMAD3 or SMAD7 or SMARCA4 or SMARCE1 or SOCAR or SOCS3 or SPRR1B or SPRR3 or SPRY2 or STAT3 or STAT6 or STEAP2-AS1 or TESPA1 or TGM3 or TH2LCRR or THEMIS or TLR1 or TNFRSF11A or TNFRSF6B or TNXB or TRAF3 or TRIB1 or TRPM8 or TSBP1 or TSBP1-AS1 or WDR36 or XIRP2 or XIRP2-AS1 or Y_RNA or ZBTB38 or ZGLP1 or ZNF217 or ZNF365 or ZNF652 or OLFM1 or FCN1 or CDSN or CTLA-4 or DOCK8 or DSG1 or DSP or FOXP3 or IFNGR1 or IL4RA or IVL or KRT10 or MALT1 or PGM3 or RAG1 or RAG2 or SPINK5 or TARC or TSLP).mp. | 200883 |
| 15 | 13 or 14 | 205078 |
| 16 | Dermatitis, Atopic/ or exp Eczema/ or exp Asthma/ or exp Food Hypersensitivity/ or exp Hypersensitivity/ | 377806 |
| 17 | ("atopic* dermatitis" or asthma* or atopy or allerg* or wheez* or eczema).mp. | 424995 |
| 18 | 16 or 17 | 530123 |
| 19 | 4 and 15 and 18 | 2438 |

**Emcare via Ovid**

**31.03.2023**

| 1 | exp child/ or exp juvenile/ or exp adolescent/ | 782272 |
| --- | --- | --- |
| 2 | exp newborn/ or exp infant/ | 173333 |
| 3 | (infant* or newborn or neonat* or neo-nat* or baby or babies or child* or toddler* or pre?school* or pediatri* or paediatri* or adolescen* or teenage* or juvenile*).mp. | 1214373 |
| 4 | 1 or 2 or 3 | 1225084 |
| 5 | exp gene/ or "genetic association"/ or exp "human genetics"/ or "genetic variability"/ or "genetic susceptibility"/ or "genetic marker"/ or "genetic analysis"/ or exp DNA/ | 221494 |
| 6 | (gene* or genome* or exome* or exon or Transcriptome* or loci or "Single Nucleotide Polymorphism" or "Deoxyribonucleic acid" or DNA or Chromosom*).mp. | 1566516 |
| 7 | 5 or 6 | 1568470 |
| 8 | ((skin or epidermal) adj4 barrier*).mp. | 1726 |
| 9 | ("transepidermal water loss" or TEWL).mp. | 621 |
| 10 | "Stratum corneum hydration".mp. | 84 |
| 11 | "skin permeability"/ or "skin barrier dysfunction"/ or "skin allergy"/ or "skin sensitization"/ | 2722 |
| 12 | 8 or 9 or 10 or 11 | 4673 |
| 13 | 7 and 12 | 1126 |
| 14 | (ACTL9 or ADO or AFF1 or AIMP1P2 or ALDH7A1P4 or ANKRD11P1 or AOAH or AP5B1 or ATP6V1G2-DDX39B or BCLAF1P1 or BHLHE40 or C3orf35 or CAPSL or CARD11 or CAVIN1 or CCDC80 or CCR7 or CD207 or CD247 or CDH13 or CHCHD3P1 or CHROMR or CHST8 or CIART or CLEC16A or CPE or CRCT1 or CREB5 or CSF2RB or CSGALNACT1 or CTIF or CTNNA2 or CYP24A1 or D2HGDH or DCHS2 or DDX39B or DMRTA1 or EEF1A1P27 or EFHC1 or EGR2 or EHMT1 or EMSY or ERBB3 or ERG or ETS1 or EVI5 or FAM177A1 or FGF20 or *FLG* or *FLG*-AS1 or FOXCUT or GAPDHP41 or GLB1 or GOLGA4 or GPHN or GPSM3 or GRM4 or HLA-B or HLA-DQA1 or HLA-DRB1 or HMSD or IKZF3 or IL13 or IL15RA or *IL18*R1 or *IL18*RAP or IL1R1 or IL1RL1 or IL21 or IL22 or IL2RA or IL6R or IL7R or INPP5D or IQGAP1 or IRAK1BP1 or ITGB8 or KCND2 or KCTD8 or KIAA1109 or KIF3A or KPNA3 or KRT8P26 or KRT8P37 or LARP7P4 or LAYN or LCE1E or LCE1F or LCE3A or LCE3E or LCE5A or LCEP4 or LGMNP1 or LINC00299 or LINC00603 or LINC00624 or LINC00709 or LINC00824 or LINC00861 or LINC01080 or LINC01143 or LINC01503 or LINC01653 or LINC01882 or LINC01993 or LINC02042 or LINC02055 or LINC02098 or LINC02571 or LINC02676 or LINC02757 or LRRC32 or MDM1 or MEI4 or MFN2 or MGMT or MICB or MIR302F or MIR4435-2HG or MIR5708 or MRPS21 or MSL3P1 or MTND5P15 or MYT1L or NAA35 or NBAS or NBPF17P or NCF4 or NCF4-AS1 or NCKAP5 or NFILZ or NLRP10 or NONOP2 or OR10A3 or 2W5P or OVOL1 or PA2G4 or PBX2 or PFDN4 or PGLYRP4 or PHB or PHBP7 or PON1 or PPP2R3C or PRKRA or PRORP or PRR5L or PTPN2 or PTPRN2 or PUDPP2 or PUS10 or R3HCC1L or RAD50 or RASIP1 or RBBP4P4 or RBMS3 or RCBTB1 or REL or RN7SL335P or RN7SL352P or RN7SL474P or RNA5SP144 or RNF111 or RNU1-141P or RNU6-1213P or RNU6-408P or RPL21P17 or RPL23AP39 or RPL41 or RPS3AP21 or RSL24D1 or RTEL1 or RTEL1-TNFRSF6B or S100A11 or SCAPER or SDR42E1P5 or SEMA6C or SERPINB10 or SFRP2 or SIK2 or SLC25A38P1 or SLC25A46 or SLC9A4 or SMAD3 or SMAD7 or SMARCA4 or SMARCE1 or SOCAR or SOCS3 or SPRR1B or SPRR3 or SPRY2 or STAT3 or STAT6 or STEAP2-AS1 or TESPA1 or TGM3 or TH2LCRR or THEMIS or TLR1 or TNFRSF11A or TNFRSF6B or TNXB or TRAF3 or TRIB1 or TRPM8 or TSBP1 or TSBP1-AS1 or WDR36 or XIRP2 or XIRP2-AS1 or Y_RNA or ZBTB38 or ZGLP1 or ZNF217 or ZNF365 or ZNF652 or OLFM1 or FCN1 or CDSN or CTLA-4 or DOCK8 or DSG1 or DSP or FOXP3 or IFNGR1 or IL4RA or IVL or KRT10 or MALT1 or PGM3 or RAG1 or RAG2 or SPINK5 or TARC or TSLP).mp. | 31732 |
| 15 | 13 or 14 | 32754 |
| 16 | exp "atopic dermatitis"/ or exp eczema/ or exp asthma/ or exp allergic asthma/ or exp atopy/ or exp food allergy/ or exp skin allergy/ or allergy/ or exp wheezing/ | 65100 |
| 17 | ("atopic* dermatitis" or asthma* or atopy or allerg* or wheez* or eczema).mp. | 110013 |
| 18 | 16 or 17 | 110160 |
| 19 | 4 and 15 and 18 | 460 |

**CINAHL via Ebsco**

**31.03.2023**

| S1 | (MH "Child+") OR (MH "Adolescence+") | 1,105,971 |
| --- | --- | --- |
| S2 | TI ( infant* or newborn or neonat* or neo-nat* or baby or babies or child* or toddler* or preschool* or pre-school* or pediatri* or paediatri* or adolescen* or teenage* or juvenile* ) OR AB ( infant* or newborn or neonat* or neo-nat* or baby or babies or child* or toddler* or preschool* or pre-school* or pediatri* or paediatri* or adolescen* or teenage* or juvenile* ) | 925,097 |
| S3 | S1 OR S2 | 1,404,904 |
| S4 | (MH "Genetics+") | 229,918 |
| S5 | (MH "Genetic Markers") | 4,259 |
| S6 | (MH "DNA+") | 35,407 |
| S7 | TI ( gene* or genome* or exome* or exon or Transcriptome* or loci or "Single Nucleotide Polymorphism" or "Deoxyribonucleic acid" or DNA or Chromosom* ) OR AB ( gene* or genome* or exome* or exon or Transcriptome* or loci or "Single Nucleotide Polymorphism" or "Deoxyribonucleic acid" or DNA or Chromosom* ) | 794,915 |
| S8 | S4 OR S5 OR S6 OR S7 | 893,808 |
| S9 | TI ( (skin or epidermal) N4 barrier* ) OR AB ( (skin or epidermal) N4 barrier* ) | 1,252 |
| S10 | TI ( "transepidermal water loss" or TEWL ) OR AB ( "transepidermal water loss" or TEWL ) | 318 |
| S11 | TI "Stratum corneum hydration" OR AB "Stratum corneum hydration" | 54 |
| S12 | (MH "Water Loss, Insensible") | 150 |
| S13 | S9 OR S10 OR S11 OR S12 | 1,518 |
| S14 | S8 AND S13 | 330 |
| S15 | TI ( ACTL9 or ADO or AFF1 or AIMP1P2 or ALDH7A1P4 or ANKRD11P1 or AOAH or AP5B1 or ATP6V1G2-DDX39B or BCLAF1P1 or BHLHE40 or C3orf35 or CAPSL or CARD11 or CAVIN1 or CCDC80 or CCR7 or CD207 or CD247 or CDH13 or CHCHD3P1 or CHROMR or CHST8 or CIART or CLEC16A or CPE or CRCT1 or CREB5 or CSF2RB or CSGALNACT1 or CTIF or CTNNA2 or CYP24A1 or D2HGDH or DCHS2 or DDX39B or DMRTA1 or EEF1A1P27 or EFHC1 or EGR2 or EHMT1 or EMSY or ERBB3 or ERG or ETS1 or EVI5 or FAM177A1 or FGF20 or *FLG* or *FLG*-AS1 or FOXCUT or GAPDHP41 or GLB1 or GOLGA4 or GPHN or GPSM3 or GRM4 or HLA-B or HLA-DQA1 or HLA-DRB1 or HMSD or IKZF3 or IL13 or IL15RA or *IL18*R1 or *IL18*RAP or IL1R1 or IL1RL1 or IL21 or IL22 or IL2RA or IL6R or IL7R or INPP5D or IQGAP1 or IRAK1BP1 or ITGB8 or KCND2 or KCTD8 or KIAA1109 or KIF3A or KPNA3 or KRT8P26 or KRT8P37 or LARP7P4 or LAYN or LCE1E or LCE1F or LCE3A or LCE3E or LCE5A or LCEP4 or LGMNP1 or LINC00299 or LINC00603 or LINC00624 or LINC00709 or LINC00824 or LINC00861 or LINC01080 or LINC01143 or LINC01503 or LINC01653 or LINC01882 or LINC01993 or LINC02042 or LINC02055 or LINC02098 or LINC02571 or LINC02676 or LINC02757 or LRRC32 or MDM1 or MEI4 or MFN2 or MGMT or MICB or MIR302F or MIR4435-2HG or MIR5708 or MRPS21 or MSL3P1 or MTND5P15 or MYT1L or NAA35 or NBAS or NBPF17P or NCF4 or NCF4-AS1 or NCKAP5 or NFILZ or NLRP10 or NONOP2 or OR10A3 or 2W5P or OVOL1 or PA2G4 or PBX2 or PFDN4 or PGLYRP4 or PHB or PHBP7 or PON1 or PPP2R3C or PRKRA or PRORP or PRR5L or PTPN2 or PTPRN2 or PUDPP2 or PUS10 or R3HCC1L or RAD50 or RASIP1 or RBBP4P4 or RBMS3 or RCBTB1 or REL or RN7SL335P or RN7SL352P or RN7SL474P or RNA5SP144 or RNF111 or RNU1-141P or RNU6-1213P or RNU6-408P or RPL21P17 or RPL23AP39 or RPL41 or RPS3AP21 or RSL24D1 or RTEL1 or RTEL1-TNFRSF6B or S100A11 or SCAPER or SDR42E1P5 or SEMA6C or SERPINB10 or SFRP2 or SIK2 or SLC25A38P1 or SLC25A46 or SLC9A4 or SMAD3 or SMAD7 or SMARCA4 or SMARCE1 or SOCAR or SOCS3 or SPRR1B or SPRR3 or SPRY2 or STAT3 or STAT6 or STEAP2-AS1 or TESPA1 or TGM3 or TH2LCRR or THEMIS or TLR1 or TNFRSF11A or TNFRSF6B or TNXB or TRAF3 or TRIB1 or TRPM8 or TSBP1 or TSBP1-AS1 or WDR36 or XIRP2 or XIRP2-AS1 or Y_RNA or ZBTB38 or ZGLP1 or ZNF217 or ZNF365 or ZNF652 or OLFM1 or FCN1 or CDSN or CTLA-4 or DOCK8 or DSG1 or DSP or FOXP3 or IFNGR1 or IL4RA or IVL or KRT10 or MALT1 or PGM3 or RAG1 or RAG2 or SPINK5 or TARC or TSLP ) OR AB ( ACTL9 or ADO or AFF1 or AIMP1P2 or ALDH7A1P4 or ANKRD11P1 or AOAH or AP5B1 or ATP6V1G2-DDX39B or BCLAF1P1 or BHLHE40 or C3orf35 or CAPSL or CARD11 or CAVIN1 or CCDC80 or CCR7 or CD207 or CD247 or CDH13 or CHCHD3P1 or CHROMR or CHST8 or CIART or CLEC16A or CPE or CRCT1 or CREB5 or CSF2RB or CSGALNACT1 or CTIF or CTNNA2 or CYP24A1 or D2HGDH or DCHS2 or DDX39B or DMRTA1 or EEF1A1P27 or EFHC1 or EGR2 or EHMT1 or EMSY or ERBB3 or ERG or ETS1 or EVI5 or FAM177A1 or FGF20 or *FLG* or *FLG*-AS1 or FOXCUT or GAPDHP41 or GLB1 or GOLGA4 or GPHN or GPSM3 or GRM4 or HLA-B or HLA-DQA1 or HLA-DRB1 or HMSD or IKZF3 or IL13 or IL15RA or *IL18*R1 or *IL18*RAP or IL1R1 or IL1RL1 or IL21 or IL22 or IL2RA or IL6R or IL7R or INPP5D or IQGAP1 or IRAK1BP1 or ITGB8 or KCND2 or KCTD8 or KIAA1109 or KIF3A or KPNA3 or KRT8P26 or KRT8P37 or LARP7P4 or LAYN or LCE1E or LCE1F or LCE3A or LCE3E or LCE5A or LCEP4 or LGMNP1 or LINC00299 or LINC00603 or LINC00624 or LINC00709 or LINC00824 or LINC00861 or LINC01080 or LINC01143 or LINC01503 or LINC01653 or LINC01882 or LINC01993 or LINC02042 or LINC02055 or LINC02098 or LINC02571 or LINC02676 or LINC02757 or LRRC32 or MDM1 or MEI4 or MFN2 or MGMT or MICB or MIR302F or MIR4435-2HG or MIR5708 or MRPS21 or MSL3P1 or MTND5P15 or MYT1L or NAA35 or NBAS or NBPF17P or NCF4 or NCF4-AS1 or NCKAP5 or NFILZ or NLRP10 or NONOP2 or OR10A3 or 2W5P or OVOL1 or PA2G4 or PBX2 or PFDN4 or PGLYRP4 or PHB or PHBP7 or PON1 or PPP2R3C or PRKRA or PRORP or PRR5L or PTPN2 or PTPRN2 or PUDPP2 or PUS10 or R3HCC1L or RAD50 or RASIP1 or RBBP4P4 or RBMS3 or RCBTB1 or REL or RN7SL335P or RN7SL352P or RN7SL474P or RNA5SP144 or RNF111 or RNU1-141P or RNU6-1213P or RNU6-408P or RPL21P17 or RPL23AP39 or RPL41 or RPS3AP21 or RSL24D1 or RTEL1 or RTEL1-TNFRSF6B or S100A11 or SCAPER or SDR42E1P5 or SEMA6C or SERPINB10 or SFRP2 or SIK2 or SLC25A38P1 or SLC25A46 or SLC9A4 or SMAD3 or SMAD7 or SMARCA4 or SMARCE1 or SOCAR or SOCS3 or SPRR1B or SPRR3 or SPRY2 or STAT3 or STAT6 or STEAP2-AS1 or TESPA1 or TGM3 or TH2LCRR or THEMIS or TLR1 or TNFRSF11A or TNFRSF6B or TNXB or TRAF3 or TRIB1 or TRPM8 or TSBP1 or TSBP1-AS1 or WDR36 or XIRP2 or XIRP2-AS1 or Y_RNA or ZBTB38 or ZGLP1 or ZNF217 or ZNF365 or ZNF652 or OLFM1 or FCN1 or CDSN or CTLA-4 or DOCK8 or DSG1 or DSP or FOXP3 or IFNGR1 or IL4RA or IVL or KRT10 or MALT1 or PGM3 or RAG1 or RAG2 or SPINK5 or TARC or TSLP ) | 93,688 |
| S16 | S14 OR S15 | 93,980 |
| S17 | (MH "Hypersensitivity+") | 77,856 |
| S18 | (MH "Eczema") | 2,797 |
| S19 | (MH "Asthma+") | 38,170 |
| S20 | TI ( "atopic* dermatitis" or asthma* or atopy or allerg* or wheez* or eczema ) OR AB ( "atopic* dermatitis" or asthma* or atopy or allerg* or wheez* or eczema ) | 74,770 |
| S21 | S17 OR S18 OR S19 OR S20 | 104,281 |
| S22 | S3 AND S16 AND S21 | 444 |

**Cochrane via Wiley**

**31.03.2023**

| #1 | MeSH descriptor: [Child] explode all trees | 77474 |
| --- | --- | --- |
| #2 | MeSH descriptor: [Adolescent] this term only | 125164 |
| #3 | MeSH descriptor: [Infant] explode all trees | 41435 |
| #4 | (infant* or newborn or neonat* or neo-nat* or baby or babies or child* or toddler* or preschool* or pre-school* or pediatri* or paediatri* or adolescen* or teenage* or juvenile*):ti,ab,kw | 343393 |
| #5 | {OR #1-#4} | 343393 |
| #6 | MeSH descriptor: [Genes] explode all trees | 2275 |
| #7 | MeSH descriptor: [Genetic Predisposition to Disease] explode all trees | 1596 |
| #8 | MeSH descriptor: [Genetics] explode all trees | 2043 |
| #9 | MeSH descriptor: [Genetic Variation] explode all trees | 8985 |
| #10 | MeSH descriptor: [Genetic Markers] this term only | 354 |
| #11 | MeSH descriptor: [DNA] explode all trees | 2861 |
| #12 | (gene* or genome* or exome* or exon or Transcriptome* or loci or "Single Nucleotide Polymorphism" or "Deoxyribonucleic acid" or DNA or Chromosom*):ti,ab,kw | 289381 |
| #13 | {OR #6-#12} | 290260 |
| #14 | ((skin or epidermal) near/4 barrier*):ti,ab,kw | 1155 |
| #15 | ("transepidermal water loss" or TEWL):ti,ab,kw | 1470 |
| #16 | ("Stratum corneum hydration"):ti,ab,kw | 159 |
| #17 | MeSH descriptor: [Skin Absorption] this term only | 319 |
| #18 | {OR #14-#17} | 2362 |
| #19 | #13 and #18 | 353 |
| #20 | (ACTL9 or ADO or AFF1 or AIMP1P2 or ALDH7A1P4 or ANKRD11P1 or AOAH or AP5B1 or "ATP6V1G2 DDX39B" or BCLAF1P1 or BHLHE40 or C3orf35 or CAPSL or CARD11 or CAVIN1 or CCDC80 or CCR7 or CD207 or CD247 or CDH13 or CHCHD3P1 or CHROMR or CHST8 or CIART or CLEC16A or CPE or CRCT1 or CREB5 or CSF2RB or CSGALNACT1 or CTIF or CTNNA2 or CYP24A1 or D2HGDH or DCHS2 or DDX39B or DMRTA1 or EEF1A1P27 or EFHC1 or EGR2 or EHMT1 or EMSY or ERBB3 or ERG or ETS1 or EVI5 or FAM177A1 or FGF20 or *FLG* or "*FLG* AS1" or FOXCUT or GAPDHP41 or GLB1 or GOLGA4 or GPHN or GPSM3 or GRM4 or "HLA B" or "HLA DQA1" or "HLA DRB1" or HMSD or IKZF3 or IL13 or IL15RA or *IL18*R1 or *IL18*RAP or IL1R1 or IL1RL1 or IL21 or IL22 or IL2RA or IL6R or IL7R or INPP5D or IQGAP1 or IRAK1BP1 or ITGB8 or KCND2 or KCTD8 or KIAA1109 or KIF3A or KPNA3 or KRT8P26 or KRT8P37 or LARP7P4 or LAYN or LCE1E or LCE1F or LCE3A or LCE3E or LCE5A or LCEP4 or LGMNP1 or LINC00299 or LINC00603 or LINC00624 or LINC00709 or LINC00824 or LINC00861 or LINC01080 or LINC01143 or LINC01503 or LINC01653 or LINC01882 or LINC01993 or LINC02042 or LINC02055 or LINC02098 or LINC02571 or LINC02676 or LINC02757 or LRRC32 or MDM1 or MEI4 or MFN2 or MGMT or MICB or MIR302F or "MIR4435 2HG" or MIR5708 or MRPS21 or MSL3P1 or MTND5P15 or MYT1L or NAA35 or NBAS or NBPF17P or NCF4 or "NCF4 AS1" or NCKAP5 or NFILZ or NLRP10 or NONOP2 or OR10A3 or 2W5P or OVOL1 or PA2G4 or PBX2 or PFDN4 or PGLYRP4 or PHB or PHBP7 or PON1 or PPP2R3C or PRKRA or PRORP or PRR5L or PTPN2 or PTPRN2 or PUDPP2 or PUS10 or R3HCC1L or RAD50 or RASIP1 or RBBP4P4 or RBMS3 or RCBTB1 or REL or RN7SL335P or RN7SL352P or RN7SL474P or RNA5SP144 or RNF111 or "RNU1 141P" or "RNU6 1213P" or "RNU6 408P" or RPL21P17 or RPL23AP39 or RPL41 or RPS3AP21 or RSL24D1 or RTEL1 or "RTEL1 TNFRSF6B" or S100A11 or SCAPER or SDR42E1P5 or SEMA6C or SERPINB10 or SFRP2 or SIK2 or SLC25A38P1 or SLC25A46 or SLC9A4 or SMAD3 or SMAD7 or SMARCA4 or SMARCE1 or SOCAR or SOCS3 or SPRR1B or SPRR3 or SPRY2 or STAT3 or STAT6 or "STEAP2 AS1" or TESPA1 or TGM3 or TH2LCRR or THEMIS or TLR1 or TNFRSF11A or TNFRSF6B or TNXB or TRAF3 or TRIB1 or TRPM8 or TSBP1 or "TSBP1 AS1" or WDR36 or XIRP2 or "XIRP2 AS1" or "Y RNA" or ZBTB38 or ZGLP1 or ZNF217 or ZNF365 or ZNF652 or OLFM1 or FCN1 or CDSN or "CTLA 4" or DOCK8 or DSG1 or DSP or FOXP3 or IFNGR1 or IL4RA or IVL or KRT10 or MALT1 or PGM3 or RAG1 or RAG2 or SPINK5 or TARC or TSLP):ti,ab,kw | 5522 |
| #21 | #19 or #20 | 5844 |
| #22 | MeSH descriptor: [Dermatitis, Atopic] this term only | 2304 |
| #23 | MeSH descriptor: [Eczema] explode all trees | 1487 |
| #24 | MeSH descriptor: [Asthma] explode all trees | 14942 |
| #25 | MeSH descriptor: [Food Hypersensitivity] explode all trees | 1209 |
| #26 | MeSH descriptor: [Hypersensitivity] explode all trees | 25923 |
| #27 | ("atopic* dermatitis" or asthma* or atopy or allerg* or wheez* or eczema):ti,ab,kw | 67669 |
| #28 | {OR #22-#27} | 70554 |
| #29 | #5 AND #21 AND #28 | 273 |
| #30 | #29 in Cochrane Reviews | 4 |
| #31 | #29 in Trials | 269 |

**Quality Assessment**

The quality of included texts was assessed independently by IH and KP. During this process, a seven point Likert scale was used to assess eleven domains, including study rationale, identifying sources of bias, and sample size / study power, giving a total score of 77.

Each study was allocated a score, with studies without a control group corresponding to poor quality studies [≤32], moderate quality [>32 and ≤40] or good quality studies [>40], as per the Q-Genie scoring system_[27]_. Studies rated as moderate- or good-quality were included in our systematic review and meta-analysis, whilst those rated as poor-quality were excluded. In the event of inter-reviewer disagreement, CH acted as third reviewer and a consensus decision was made.

**Data extraction**

From each included full-text, the following data were extracted:

- Study details: authors, year of publication, study design (cohort vs case-control), study location
- Sample: size, age range
- Independent variable: polymorphism

For the purpose of this review, it was ascertained that polymorphisms within a single gene could be combined to gain a single estimate of effect for each gene studied

**Supplementary Results**

**Included studies**

Initially, 8633 records published between 1946 and 31 March 2023, were identified by our systematic search. This was reduced to 6018 records after de-duplication. 5060 studies were excluded at title-abstract screening, resulting in 958 records proceeding to full-text screening [Figure s1 in Supplementary Material].

**Figure s1: PRISMA for Systematic Review and Meta Analysis of Skin Barrier Related Genes in Childhood Allergic Disease**


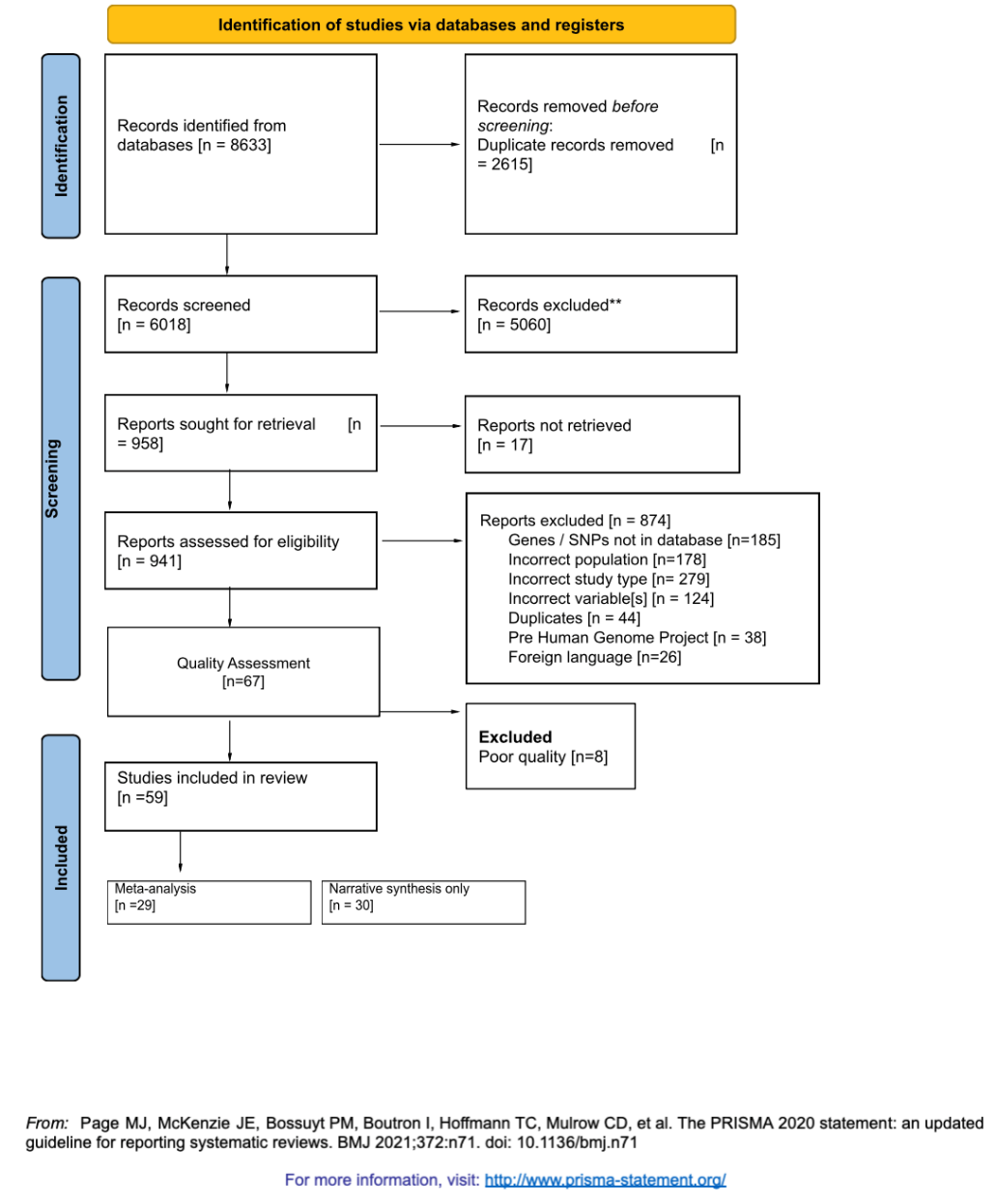


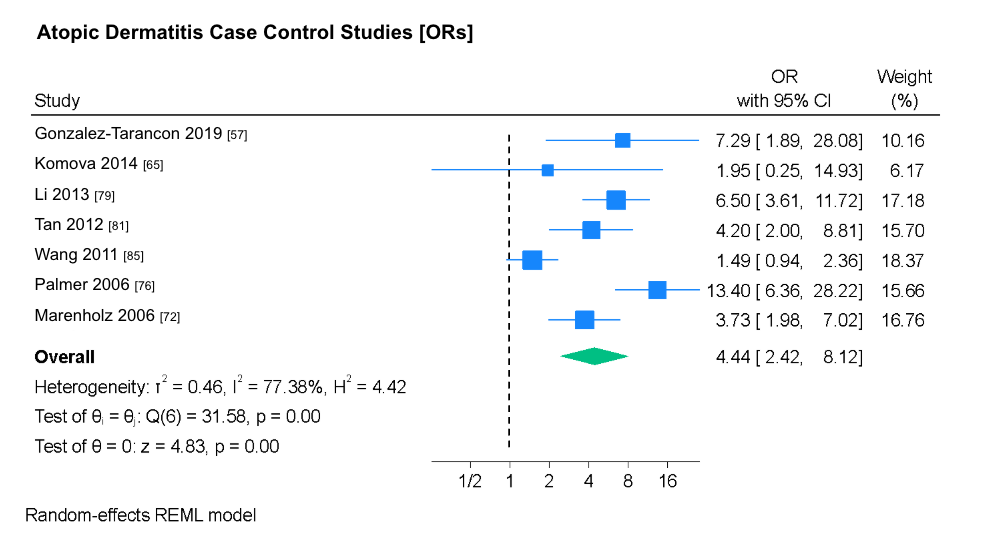
***FLG* and AD** (**case-control)**

Seven case-control studies _[57, 65, 70, 72, 77, 81, 85]_, with five showing significant associations between *FLG* null mutations and AD [Figure s2]. The pooled effect size was large [OR 4.44, 95% CI 2.42 - 8.12], with significant study heterogeneity [I^2^= 77.38%, p = <0.05].

***FLG* and AD** (**cohort)**


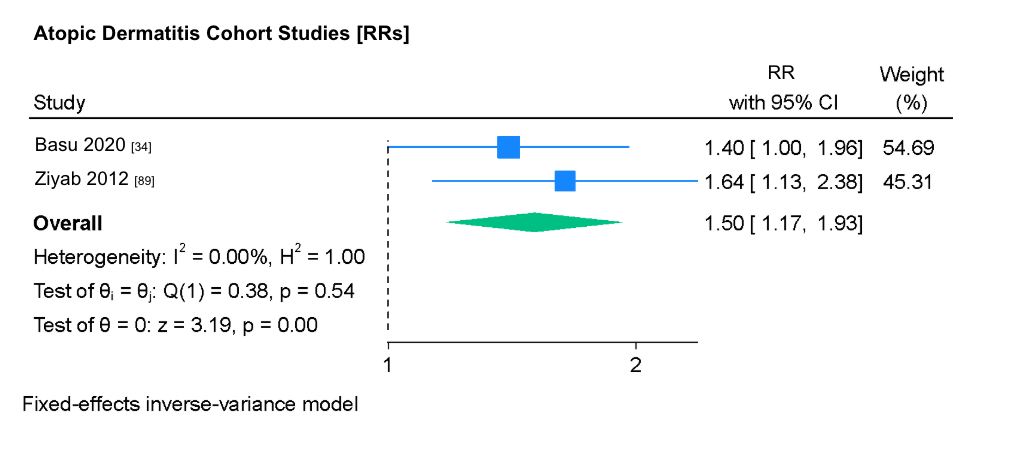
Two AD cohort studies with risk ratio outcomes_[35, 89]_ were meta-analysed [Figure s3], a significant pooled effect size [OR 1.50, 95% CI 1.17 – 1.93]**.** Heterogeneity analysis demonstrated I^2^ = 0% [p = 0.54].

**Narrative Synthesis**

**AD**

23 studies assessed outcomes related to atopic dermatitis, 22 of which concerned *FLG.* The proportion of *FLG* null mutation carriers was greater in children with AD than in healthy controls in a population-based cohort in northwest England (18.4% vs. 12.9% respectively [p=0.00012])_[42]_ in the ODENSE cohort(11% vs 5% respectively [p=0.041]_[36]_, and in a case-control study in Korea (2.6% vs 0.0% respectively [p<0.001]in Korea_[88]_. Conversely, a Turkish case-control study did not find any *FLG* null mutations amongst its case or control groups, however the study solely genotyped one mutation R501X_[52]_. This was replicated in a further Turkish case-control study; authors felt this could be partially attributed to the low frequencies of individuals with any of the four genotyped *FLG* LOF mutations (R501X, 2282del4, R2447X and S3247X)_[82]_. Additionally, in a Korean nested case-control study, there was no significant difference found in genotyped *FLG* polymorphisms 3321delA nor K4033X between children with early onset AD (n=28) and controls (n=57). These results may differ from other studies partially due to the small sample size and selection of genotyped polymorphisms_[62]_.

In the majority of studies, associations were demonstrated between *FLG* null mutations and presence of AD in childhood. In the aforementioned English population-based cohort, carriage of 2 *FLG* null mutations was associated with increased odds of childhood AD [OR 26.9 (95% CI 3.3-217.1)], whereas carriage of a single mutation was not [OR 1.2 (0.7-1.9)]_[42]_. This was replicated in the Danish COPSAC birth cohort [HR 2.23 (95% CI 1.47-3.39) p=0.0002]_[45]_.

Certain studies investigated the interaction of *FLG* polymorphisms and AD onset age, with early-onset AD universally defined as onset prior to 2-years of age. A cross-sectional analysis of Singaporean Chinese children with AD reported significantly greater odds of early-onset AD in those carrying at least one *FLG* null mutation [OR 5.6 (95% CI 1.32-4.72)]_[45]_. Another cross-sectional study of AD patients from the USA PEER study demonstrated significantly increased likelihood of early-onset AD in children with 1 *FLG* null mutation [OR 1.74 (1.02-2.96)], however this was not statistically significant in children with 2 *FLG* null mutations [OR 3.03 (0.70-13.16])_[84]_.

With respect to individual polymorphisms, one study found that presence of rs6683167 [OR 0.346 (0.03-0.49) p=0.399] and rs4126714 [OR 1.5 (1.05-2.38) p=0.014] were modestly associated with AD onset before 2-years of age in 25 children with AD_[30]_. No associations were found between rs1158340, Novel-a, rs14257422, rs2011331, rs41267154, rs66831674, Novel-b or rs3126074, and early-onset of AD. Differences in study findings may be attributed to its small sample size. Additionally, in a case-control study conducted in Italy, correlation was found between rs1131365 [p<0.02], rs3749104 [p<0.01], rs1509578 [p<0.01], rs1839422 [p<0.01] and AD onset before 3-months old. No correlation was found between rs1158434, rs7470051, rs1924558, rs1380552, rs1474721, and early-onset AD. Correlation was found between rs1458280 [p<0.01], rs1442093 [p<0.01] and rs5582691 [p<0.01] and onset between 3-6 months of age. Correlation was found between rs1158434 [p<0.01], rs3749104 [p<0.01], rs1456277 [p<0.01], rs7412944 [p<0.01] and onset after 6-months old. No correlation was found between rs7470051, rs1380552 and onset after 6-months old_[71]_.

Six studies analysed differences in eczema severity with respect to *FLG* null mutations, of which four utilised Scoring Atopic Dermatitis (SCORAD) criteria to grade eczema severity. In the Swedish BAMSE birth cohort, there was no significant difference in proportion of *FLG* mutations in mild AD versus moderate-severe AD [p=0.907]_[33]_ nor in newly-diagnosed children in Slovenia [p=0.08]_[36]_. Additionally, there was no significant difference in median SCORAD between children with *FLG* null mutations and those without in a Japanese cross-sectional analysis of children with AD_[56]_. Conversely, in English schoolchildren, those with *FLG* LOF mutations reported more severe eczema [p=0.0042], however the mean difference was of small magnitude (1-2 points in SCORAD)_[43]_. Similar findings were reported in Singaporean Chinese schoolchildren with AD, where those carrying at least 1 *FLG* mutations mutation were significantly more likely to develop severe AD, as per SCORAD criteria, compared with those carrying only wild-type, after adjusting for age and sex_[45]_. A cross-sectional sample from the Japanese PACI and PACI-ON cohorts graded eczema severity with the Eczema Area and Severity Index (EASI), and reported significantly higher median EASI in children carrying any of ten most common *FLG* LOF mutations in the Japanese population versus those carrying only wild-type [p=0.0498]_[69]_. Moreover, the COPSAC study demonstrated significant association between *FLG* null mutations and ‘steroid days’, measuring use of topical steroids in the first three years of life, but not with ‘AD days’, denoting parent-reported daily symptoms of AD, with and without adjustment for age of onset_[78]_. The study reports that COPSAC physicians validated parent-reported symptoms together within the study, yet it is important to note that, to knowledge, there was no prior validation for either scoring method_[78]_.

Regarding specific polymorphisms and AD severity, in rs1456277 [p<0.01] rs798083 [p<0.01], rs1509578 [p<0.01], rs1458280 [p<0.01] were associated with moderate AD and rs7470051 was associated with severe AD [p<0.01. Rs3749104 [p<0.01], rs1380552[p<0.01], rs1839422 [p<0.01], rs1158434 [p<0.01] and rs1131365 [p<0.01]were associated with moderate and severe AD_[71]_. In the PEER cohort of children with AD, the known null mutations R501X [OR 0.58 (0.37-0.82) p<0.0001], 2282del2 [OR 0.58 (0.40-0.82) p<0.0001], R2247X [OR 0.47 (0.24-0.91) p<0.0001] were associated with absence of symptom-free periods, whereas S4347X [OR 0.77 (0.41-1.45) p] was not. However, when analysed together, having any of the four null *FLG* mutations was associated with absence of symptom free periods [OR 0.54 (0.41-0.71) p<0.0001], and therefore persistent AD_[73]_. This was replicated when less common *FLG* exon 3 mutations variants were sequenced and also associated with absence of symptom-free periods_[73]_, and thus persistent AD[OR 0.36 (0.14-0.89) p=0.027]. Moreover, in a Korean case-control study, there was no significant association between presence of 3321delA mutation and AD severity [p=0.842]. Analyses between AD severity and other genotyped mutations were not performed in this study_[88]_.

One case-control study genotyped *FLG-2* polymorphism rs12568784 in Polish children and found significantly increased likelihood of childhood AD in children carrying this polymorphism when analysed with the allelic model of inheritance [OR 1.91 (95%CI 1.01-3.65) p=0.003], but not with the dominant model [OR 2.17 (95%CI 0.99-4.81) p=0.003]_[50]_.

**Asthma and wheeze**

Four studies investigated association between *FLG* LOF mutations and presence of asthma, with conflicting results_[31][42][52][53]_. In a cohort of Ukrainian children, the C/T genotype of *FLG* rs7927894 was associated with significantly higher odds of asthma, compared with healthy controls_[50]_. Additionally, although not a specific outcome measure for this review, they also found a significantly higher proportion of the C/T genotype of *FLG* rs7927894 in patients with both asthma and AD, compared with healthy controls. Interestingly, in an Italian cross-sectional analysis of children with AD, only two (R501X 2282del4) of ten sequenced loss-of-function mutations were found. However, there was not any significant difference in frequencies in children with concurrent asthma and those without_[31]_. This study did have a larger sample size than the former, however this was powered for food allergy rather than asthma. Similarly, in a population-based cohort in Northwest England, there was no difference in proportion of *FLG* null mutation carriers between children with parent-reported asthma and those without_[42]_. The fourth study investigated alleles of *FLG* null mutation R501X in Turkish children, however genotyping did not reveal any risk alleles in this cohort_[52]_.

Two studies investigated associations with respiratory symptoms, rather than standalone clinical asthma_[53][69]_. In Japanese children with AD, recruited from the PACI and PACI-ON cohorts, ten of the most common LOF mutations in the Japanese population were selected for analysis. Children with *FLG* mutations had significantly higher frequency of respiratory symptoms than those without *FLG* mutations at 1-year of age_[69]_. Another study investigated children admitted to hospital with first episode of wheeze and did not find a significant difference in genotype of *IL18* polymorphisms compared to healthy control, however, did not report specific data within their findings_[53]_.

**Food allergy**

With respect to food allergy alone, three studies associate *FLG* null mutations with sensitisation to peanuts, and therefore peanut allergy_[41][42][62]_. *FLG* LOF mutations (to include R50X1, 2282del4, R2447X and S3247X) were associated with IgE sensitisation to peanut at age-4 in the Swedish BAMSE cohort, but not at ages-8 or 16, nor to the other six food allergens tested at ages-4, 8 or 16. In sensitised children, those with *FLG* LOF mutations were more likely to be sensitised to peanuts, compared to those without mutations_[62]_. In a UK-based cohort, presence of *FLG* LOF mutations was significantly associated with both peanut sensitisation and allergy at 8 and 11 years old_[41]_. This remained the case in a mixed UK-, Ireland- and Netherlands-based cohort and, in this study, the association remained significant after adjusting for concomitant AD_[42]_.

However, in Japanese infants visiting allergy clinics, there was no association between combined *FLG* LOF mutations and IgE sensitisation to any of 5 common food allergens, which included peanuts. In fact, concerning specific polymorphisms, the G allele of *FLG* rs1933064 was inversely associated with food sensitization following adjustment for AD and combined mutations mutations_[75]_. Similarly, in a case-control study conducted in Turkey, *FLG* LOF mutations were not significantly associated with IgE-mediated allergy to at least one foodstuff_[82]_. It is important to note that the four mutations analysed are common in European populations and comprised R501X, carried by two individuals, 2282del4, carried by four individuals, R2447X, and S3247X, both of which were absent in case and control groups_[82]_. Correspondingly, a Finnish study did not find significant association between any of five common *FLG* null mutations and elevated IgE to cow’s milk protein, however the sample size of this study was relatively small_[79]_.

In children with coexistent AD, findings differ. In an Italian cohort of hospital outpatients with AD, *FLG* null mutations were found to be correlated with severe allergic reactions to food, defined as “anaphylaxis episodes and/or required the prescription of an adrenaline auto-injector” as per EAACI guidance. This was not the case with mild or moderate allergic reactions. Additionally, children with *FLG* null mutations had significantly elevated IgE antibodies to hazelnut compared to controls_[31]_. In a group of Japanese children with moderate-severe AD, there was no significant difference in egg-white-specific IgE levels between children with or without any of the eight most common *FLG* null mutations in the Japanese population_[57]_.

**Respiratory allergy**

In the BAMSE birth cohort, *FLG* LOF mutations were not associated with IgE sensitization to any of seven aeroallergens at ages-4, 8 or 16_[62]_. In children with coexistent AD, mutations in *FLG* R501X or 2282del4 were significantly associated with respiratory allergy, defined here as rhinitis with sensitisation to aeroallergens within the first seven years of life. There was no significant association between *FLG* mutations and non-respiratory allergy_[48]_. In the Japanese cohort with moderate-severe eczema, no significant difference in house dust mite-specific IgE levels was found between children with *FLG* null mutations and those without_[56]_.

With respect to specific polymorphisms, a case-control study conducted in India investigated the association between *FLG* R501X genotype and rhinitis, however all individuals in the rhinitis group had the wild-type allele, and therefore analysis could not be performed. Moreover, a case-control study based in Ukraine did not find significant association between *FLG* rs7927894 C/T nor T/T genotypes and presence of seasonal allergic rhino-conjunctivitis nor perennial allergic rhinitis. This study had a small sample size, with no power calculation reported_[51]_.

**Atopy**

The MPAACH cohort, an early-life cohort with AD, assessed association of *FLG* expression and subtypes of AD, comprising lesional skin i.e. visible disease vs non-lesional. In lesional skin, *FLG* expression positively correlated with allergic sensitization to both food and aeroallergens. This did not correlate in non-lesional skin_[37]_.

One study investigated the *FLG2* rs12568784 polymorphism and found significant association with IgE-mediated sensitisation to at least one of ten aeroallergens or ten food allergens tested in two groups of children, with and without AD_[50]_.

**Discussion**

**Respiratory allergy**

There is limited research on the relationship between *FLG* mutations and respiratory allergy, and meta-analysis of four studies did not reveal an association and there was high heterogeneity. The mixed results from studies included in narrative synthesis may have been contributed to by small sample sizes and limited analyses of individual *FLG* polymorphisms_[48][52]_.

Regarding previous meta-analyses of candidate gene association studies, a recent meta-analysis on genetic association studies and respiratory allergy investigated 23 polymorphisms, amongst 12 genes_[102]_. Of the 12 genes, only one (IL-18) was a skin-barrier-related gene. Correspondingly, none of the four polymorphisms found to have association with respiratory allergy were related to the skin-barrier. To knowledge, genome-wide meta-analyses performed in children and adults have not found association between skin-barrier related genes and respiratory allergy_[103][104]_

**Atopy**

Meta-analysis of studies combining food allergies and aeroallergens (atopy), revealed a significant pooled effect size and pronounced heterogeneity, likely reflecting variation in types of allergy, population characteristics and genetic backgrounds. Studies analysed using narrative synthesis reinforced the relationship between atopy and *FLG* null mutations. To the authors’ knowledge, as previously discussed, pre-existing reviews on the genetics of allergy separate outcomes into food allergy and atopy, rather than atopy.

**Study Characteristics**

Data was collected regarding study characteristics for all included studies; this has been summarised, with its corresponding quality assessment score, in Table s2.

**Table s2: Characteristics of Included Studies and Quality Assessment via Q-Genie**

This table illustrates main characteristics and outcome of quality assessment of all studies included (n=62) in this review _[30-91]_.

| **Study [author and publication year)]** | **Study design** | **Number of participants** | **Age [range in years, mean and standard deviation)]** | **Genotype** | **Outcome measures** | **Quality assessment [Q-Genie]** |
| --- | --- | --- | --- | --- | --- | --- |
| Arghavan 2021_[30]_ | Iranian case control | N=21 children with AD, N=25 healthy children | 0-5 years old | 8 of the most common *FLG* polymorphisms: rs11158340 Novela rs142574224 rs2011331 rs41267154 rs66831674 Novelb  rs3126074 | Age of onset of AD [< 6mo, 6- 24 months or >24 months] | Good |
| Astolfi 2021_[31]_ | Cross-sectional (AD), Bologna | 238 | < 14 years old, mean age at enrolment 8.2yrs, SD 5.1 | 10 *FLG* loss of function mutation sites [R501X, 2282del4, 3321delA, R1798X, S2554X, R2447X, S3247X, E3429X, E3603X and R3638X] - though only found in R501X and 2282del4 in this cohort | Severity of AD as measured by SCORAD and age of onset of AD  Presence of asthma  Presence of food allergies and severity as measured by oral food challenges as mild, moderate or severe | Good |
| Bager 2016_[32]_ | Danish Cohort - Danish National Birth Cohort [DNBc] | 1751 | Newborns at recruitment | 4 *FLG* mutationss: R501X, 2282del4, S3247X and R2447X | Presence of AD  Onset of AD (early onset versus. late onset) | Good |
| Ballardini 2013_[33]_ | Swedish birth cohort - BAMSE | 4089 | Enrolled at birth, surveyed at 1, 2, 4, 8 and 12 years old | 3 most common *FLG* mutations (R501X, R2447X and 2282del4) | Presence of AD at 1, 2, 4, 8 and 12 years old  Severity of AD age 12 [via BESS study], characterised via mild or mod-severe  Presence of asthma at age 1, 2, 4, 8 and 12 years | Good |
| Basu 2020_[34]_ | UK based cohort study [GO-CHILD] | 2312 | Enrolled at birth, analysed age 6, 12 and 24 months | 4 *FLG* mutations: R501X, 2282del4, S3247X, and R2447X | Presence of AD at 6, 12 and 24 months via parent reported survey, analyse via log-binomial regression  Presence of wheeze at 6, 12 and 24 months via parent reported survey, analyse via log-binomial regression  Presence of Rhinitis at 6, 12 and 24 months via parent reported survey, analyse via log-binomial regression | Good |
| Basu 2022_[35]_ | Danish case control (nested in birth cohort) - ODENSE child cohort | disease (AD): 99  control: 169 | included when participants were 7y (questionnaires at 18mo, 3y, 7y) | 6 most common *FLG* variants in Europe: R501X, 2282del4, R2447X, S3247X | Severity of AD: SCORAD | Good |
| Berce 2022_[36]_ | Cross sectional study, Slovenia | 52 | 3 months- 18 years old, with mean age of 30 months | 5 most common *FLG* mutations: R501X, 2282del4, R2447, S3247, 3702delG | Presence and severity of AD via SCORAD | Moderate |
| Biaginni Myers 2020_[37]_ | Cohort study, USA [Ohio] | 400 | 2-5 years old, age [median, IQR]: 2.3 [1.7 - 2.5] | *FLG* gene expression | AD severity measured via SCORAD [mild vs mod-severe]  Presence of food allergy: mono- vs polysensitisation [> 2 aeroallergens or food allergens] | Moderate |
| Bisgaard 2009_[38]_ | Danish birth cohort study [COPSAC] | 356 | Infants recruited at birth | 2 *FLG* mutationss: R501X, 2282Del4 | Presence of AD at 3 years  Severity of AD at 3 years: SCORAD  Presence of allergic Sensitisation at 6, 18 and 48 months | Good |
| Bohme 2012_[39]_ | Swedish birth cohort [BAMSE] | 2033 | Infants recruited at birth | 3 *FLG* mutations: R501X, R2447X and 2282del4 | Dry Skin Eczema (criteria and/or Dr diagnosis) at 1, 2, 4 and 8 years  Multinominal logistic regression models  Presence of allergic sensitisation to common allergens [IgE] | Moderate |
| Bonnelykke 2010_[40]_ | Danish Cohort [COPSAC] | 411 | Infants recruited at birth, followed up to age 5 | 2 *FLG* mutations: R501X, 2282Del4 | Presence of eczema: H&R criteria  Presence of wheeze or asthma; occurrence of acute severe asthma exacerbation at 6-monthly intervals from 0-5y  Specific IgEs | Moderate |
| Brough 2014_[41]_ | UK Cohort study | 1184 | Infants enrolled at birth | *FLG* genotype [wild type vs R501X, 2282del4, S3247X, R2447X and 3702delG loss of function mutations] | Presence of peanut sensitisation via skin prick testing, at 8 and 11 years old [wheal diameter >3mm] | Good |
| Brown 2008_[42]_ | Case control, UK | 784 | Age 7 – 9 years old | 5 most common European *FLG* variants: R501X, 2282del4, R2447X and S3247X; 3702delG | Presence of AD: parent questionnaire AND clinical assessment by a dermatologist via UK modified H&R criteria  AD severity: three-item severity score  Presence of asthma: parent questionnaire  Presence of seasonal rhinitis: parent questionnaire | Good |
| Brown 2009_[43]_ | UK Cohort study via unselected population birth cohort – North Cumbria Community Genetics Project | 792 | Age 7 – 9 years old | 6 most prevalent *FLG* null mutations (R501X, 2282del4, R2447X, S3247X, 3702delG, 3673delC) | Presence of AD: clinical assessment by dermatologist  Severity of AD: TIS score upon clinical assessment | Moderate |
| Brown 2011_[44]_ | Case-control study, U.K., Ireland and Netherlands  Replication case-control: Canada | Original study:  Cases = 71  Control = 1000  Replication:  Cases = 390  Control = 891 | UK: recruited at birth  Dutch: mean age 7.5y (range 3-14y)  Irish: mean age 10.5 (range 1-18y) | Original study: pooled analysis of R501X and 2282del4  Replication: R501X, 2282del4, R2447X, S3247X | Peanut allergy: positive food challenge, or clinical history and skinprick test wheal  ≥8mm and/or peanut-specific IgE  ≥15kU/L | Good |
| Cai 2012_[45]_ | Cohort study, Singapore | 228 | Mean age 10.8 years [SD 5.3yrs] | *FLG* mutation [for 22 population-specific *FLG* null mutations* vs wildtype --> categorised as null vs non-null] *R501X 3321delA 441delA G323X Q368X S406X 1249insG S1302X 1640delG 2952delC 4275del2 S1515X Q1745X 6950_6957del8 8393delA 9040_9058dup Q2417X E2422X 7945delA S2706X R4307X | Age of onset of AD  Severity of AD: SCORAD index, prior episodes of disease flares, history of skin infection | Moderate |
| Carson 2012_[46]_ | COPSAC [children of mothers with asthma] birth cohort, Denmark | 397 | Newborns at enrolment | 2 most common *FLG* mutations: R501X and 2282del4 | Presence of AD: clinical diagnosis  Anatomical localisation: partial least squares discriminant analysis (PLSDA) on clinical assessment by a doctor  Severity of AD: SCORAD on clinical assessment by a doctor | Moderate |
| Chauhan 2020_[47]_ | Case control study, India | 90 | Children with AD aged 1-12, controls “school aged” | *FLG* mutation (for R501X genotypes) AA: mutant homozygous Aa: heterozygous aa: wildtype | Presence of AD: H&R criteria  Presence of asthma: GINA criteria  Presence of asthma with AD  Presence of rhinitis | Moderate |
| Chawes 2010_[48]_ | Case control, Denmark | 290 | Recruited at birth, data collection at 7y | *FLG*: carriage of 1+ null mutation (R501x or 2282del4) | Presence of respiratory allergy symptoms | Moderate |
| Debinska 2017_[49]_ | Cohort study, Poland | 158 | Recruited 0 – 2 yrs old, analysed at yearly intervals from 2-4yrs | *FLG*: carriage of 1+ loss of function mutation (from R501X, 2282del4, R2447X and S2347X) | Presence of AD: via H&R criteria and presence of visible symptoms  Severity of AD: SCORAD | Moderate |
| Debinska 2022_[50]_ | Case control, Poland | 188 | Recruited 0-2 years old  AD [mean age 13.2 ± 6.7 months] controls [mean age 15.3 ± 5.6 month] | *FLG2* rs12567874 SNP genotype (GG%, GG, GT, TT) - dominant model and allele model - G is 'normal' allele *FLG* combined genotype - based on screening for '4 common mutations': R501X, 2282del4, R2447X, S3247X | Presence of AD  Severity of AD: SCORAD  Presence of asthma  Presence of allergic sensitisation: specific IgE (to at least one of the tested allergens) of ≥0.7 kU/L (class II), or polysensitisation [>1 tested allergen] [allergens were 10 popular inhalants and 10 foods] | Good |
| Dytiatkovskyi 2019_[51]_ | Case Control, Ukraine | Cases N = 119  Control = 34 | cases [mean age: 7.9 ±0.3 year]  controls [mean age: 11.0 ±0.7 years] | 1 *FLG* SNP rs7927894 | Presence of atopic dermatitis Presence of bronchial asthma  Presence of seasonal allergic rhino-conjunctivitis or perennial respiratory allergy | Moderate |
| Ercan 2013_[52]_ | Case control, Turkey | Cases N = 49  Controls N = 50 | Cases recruited at 2mo – 16 years, cases were “age matched controls”  Cases of AD [4.9 ± 3.6 years]  Control group [3.8 ± 2.8 years] | 1 *FLG* mutation (for R501X) | Presence of AD: paediatric allergy assessment and diagnosis as per H&R criteria  AD severity: SCORAD  Presence of recurrent wheeze  Presence of asthma: GINA criteria  Severity of asthma: number of asthma attacks in the last 6mo [categorised into intermittent, mild persistent, moderate persistent and severe persistent]  Presence of respiratory allergy  Severity of respiratory allergy: ARIA criteria | Moderate |
| Esposito 2014_[53]_ | Case control, Italy | Control N = 199  Case N = 119 | Cases: infants < 12mo  Controls: healthy and age matched controls | *IL18* Rs187238 SNP | Risk of recurrent wheeze: further i.e. >1 total admission to hospital with episode of respiratory wheeze | Good |
| Flohr 2010_[54]_ | UK Cohort study | 88 | 3 month old infants | Four most common *FLG* mutations found in the U.K. white population (R501X, 2282del4, R2447X and S3247X) | Presence of atopic dermatitis at 3mo  Severity: SCORAD | Moderate |
| Flohr 2014_[55]_ | UK Cohort study | 619 | 3 month old infants | 6 most common *FLG* mutations R501X, 2282del4, R2447X, S3247X, 3673delC, and 3702del | Presence of atopic dermatitis at 3mo  Presence of any food sensitisation | Good |
| Fukuie 2019_[56]_ | Cross sectional study, Japan | N = 57 [cases] | 2mo to 5 yrs, median age: 22 months | 8 most prevalent *FLG* mutations in the Japanese population: R501X, 3321delA, S1695X, Q1701X, S2554X, S2889X, S3296X, and K4022X | Severity of AD: SCORAD  Presence of egg allergy: egg-white specific IgE  Presence of dust allergy: house dust mite-specific IgE | Good |
| Gonzalez-Tarancon 2019_[57]_ | Case control, Spain | n = 214  AD = 111 Controls = 103 | AD group mean age: 5.51 (3.93) control group mean age: 9.72 (6.22) | 3 *FLG* null mutations (R501X, 2282del4, and R2447X | Presence of AD  Severity of AD: SCORAD  Presence of asthma  Presence of rhinitis | Moderate |
| Henderson 2008_[58]_ | UK based cohort [ALPSAC] | 6971 | Enrolled at birth, analysed at 6, 18, 30, 42, 57, and 81 months of age and then annually form 7 -11yo | 2 *FLG* null alleles [R501X and 2282del4] | Presence of atopic dermatitis: parental questionnaire on skin at 6, 18, 30, 42, 57 and 81 mo with diagnosis if reported flexural dermatitis at 2 timepoints between 6 – 42mo old and clinical assessment at age 7, 9 and 11 yrs ISAAC criteria]  Presence of early wheeze: parental questionnaire at above ages  Presence of asthma: questionnaire at 91 months [doctor diagnosis plus presence of symptoms in last 12mo] | Good |
| Henneman 2015_[59]_ | European case control from EuroPrevall Cohort study | N = 20 cases  N = 23 age matched controls | Age at sampling:  Cases 11.8 ± 4.9 [months]  Controls 17.2 ± 7. [months] | *FLG* mutations: (from R501X, 2282del4, 3702delG, R1474X (did not show consistent results and was therefore excluded from analysis), 530delG, 6867delAG, E2422X, 7267delCA, R2447X, S3247X, 11029delCA, 11033del4, Q3683X ) | Presence of cow’s milk protein allergy: positive result on double-blind placebo-controlled food challenge | Good |
| Hirota 2017_[60]_ | Japanese case-control | Original study  Cases: 593  Controls: 985  Replication study  Cases: 279 Controls: 886 | Original study: Cases: mean 5.2yrs (SD 3.7yrs)  Controls: 50.0yrs (9.2yrs)  Replication study  Cases: 5.8yrs (3.3yrs)  Controls: 50.6yrs (12.9yrs) | 6 *FLG* null variants: C.3321delA, p.Q1701, p.S2554, p.S2889, p.S3296, p.K0422 | Presence of food allergy: positive oral food challenge or a clinical history after food ingestion | Moderate |
| Hoyer 2021_[61]_ | Scandinavian population based cohort study [PreventADALL] -Norway and Sweden | 1836 | Recruited at birth | 3 *FLG* mutations: R501X, 2282del4, R2447X | Presence of AD at 3, 6 and 12mo: clinical assessment and UKWP criteria ± H&R criteria >12mo | Good |
| Johansson 2017_[62]_ | Swedish cohort [BAMSE] | 1890 | Enrolled at birth, analysed at 4, 8 and 16yrs old | 3 *FLG* mutations: 2282del4, R501X and R2447X | Presence of food or respiratory allergens: IgE sensitisation, and mono- vs. oligo- [2-3] or polysensitisation [> 4] | Good |
| Kalb 2022_[63]_ | Cohort, then Case control  Germany | Cohort disease- 890 food allergy-GOFA Case-control disease- 890 food allergy-GOFA control-871 MAS (population-based) | GOFA recruited in clinics at diagnosis - mean age 2.36 (+/-2.69 SD) MAS recruited at birth | 4 most common *FLG* mutations: R501X, 2282del4, R2447X, and S3247X | Presence of eczema: physician’s diagnosis, parental report of eczema symptoms or visible eczema at the time of follow-up.  Allergy to any of ten specific foods: children with a convincing history of an immediate, allergic reaction plus specific IgE to the same food >35 kU/L | Good |
| Kim 2019_[64]_ | Case control, Korea | Cases N = 28, Control N = 57 |  | 2 *FLG* mutations: 3321delA and K4033X | Age of onset of AD | Moderate |
| Komova 2014_[65]_ | Case control, Russia | Children with AD: 140 controls: 90 | disease: 1-14y control: 3-14y | 5 *FLG* gene null mutations: 2282del4, R501X, R2447X, 3702delG, S3247X, and the 12-repeat allele (rs12730241) | Presence of AD | Moderate |
| Kono 2018_[66]_ | Japanese cohort study | 411 | 6-12yrs mean age [months] 111.1 ± 19.9 | 10 *FLG* mutations specific to Japanese population: Arg501X 3321delA Ser1695X Gln1701X Ser2554X Ser2889X Ser3296X Lys4022X Q1790X 441-442delA | Presence of AD  Presence of asthma, analysed  Presence of allergic rhinitis,  Presence of current food allergy: antigen-specific IgE testing to 6 allergens and parent questionnaire | Moderate |
| Koseki 2019_[67]_ | Japanese birth cohort study [T-CHILD] | 738 | Enrolled at birth, genotyped at 5 years, analysed at 6 months and 6yrs | 8 *FLG* mutations: c.3321del, p.Ser1695Ter, p. Gln1701Ter, p.Gln1790Ter, p.Ser2554Ter, p.Ser2889Ter, p.Ser3296Ter, and p.Lys4022Ter | Presence of AD at 6mo and at 1, 1.5, 2, 3, 4, 5, and 6yo: physician diagnosis  Onset of AD in infancy at 6mo – 2yrs and in childhood at 3-6yo  Presence of asthma at 5yo: ISAAC questionnaire,  Presence of wheeze at 5yo  Presence of respiratory allergy at 5yo | Good |
| Kotsapas 2022_[68]_ | UK Cohort (Manchester Asthma and Allergy Study) | N = 959 | Enrolled at birth, evaluated age 5 and 8 | 6 *FLG* mutations: R501X, S3247X, R2447X, 2282del4, 3673delC and 3702delG | Presence of peanut allergy: positive skinprick test or presence of peanut specific-IgE at 8yo  Presence of allergic rhinitis | Good |
| Kumagai 2023_[69]_ | PACI-ON cohort, Japan | N = 332 | Recruited at 7 – 13 weeks old | 10 most common *FLG* mutations in Japanese population: p.Arg501X c.3321delA p.Ser1695X p.Gln1701X p.Ser2554X p.Ser2889X p.Ser3296X p.Lys4022X p.Q1790X c.441-442delAG | Severity of AD at 12mo: EASI criteria and patient-orientated eczema measure [POEM]  Presence of wheeze at 12mo: symptom report  Presence of respiratory allergy at 12mo: serum IgE to specific environmental allergens | Moderate |
| Li 2013_[70]_ | Case control, China | n = 339 with AD n = 301 controls |  | *FLG* mutations [overlapping PCR strategy enabling sequencing of entire gene] | Presence of AD: clinical diagnosis and H&R criteria  Presence of AD plus respiratory allergy  Presence of AD plus asthma | Moderate |
| Manti 2017_[71]_ | Case control, Italy | Cases N = 100, controls n = 97 | AD group: 5.31years (+/- 2.33)  Control group 4.51 years (+/- 3) | Genotyping for allelic variants in the promoter and coding exon of *FLG* | Age of onset of AD: diagnosed via clinical assessment, as early onset [< 3mo], intermediate [3-6mo] and late onset [> 6mo]  Severity of AD: SCORAD | Moderate |
| Marenholz 2006_[72]_ | Cohort study - involved two study groups 1. GENUFAD [excluded] 2. German MAS cohort | 871 | Recruited at birth, analysed at 1, 3, 6, 12, 18, and 24 months, then yearly up to age 10 | 2 *FLG* mutations: R501X and 2282del4 | Presence of AD: physician diagnosis, parental report of symptoms or visible eczema at follow up  Food allergy: total and specific-IgE against 9 common food and inhalant allergens at 1, 2, 3, 5, 7 and 10yo | Good |
| Margolis 2012_[73]_ | US Cohort study [PEER] | 857 | Enrolled after the age of 2 | 4 most prevalent *FLG* null mutations: R501X, 2282del4, R2447X, and S3247X | Presence of AD: parental questionnaire based on UKWP diagnostic criteria  Severity: time measured-symptom free | Good |
| Margolis 2018_[74]_ | US Cohort study [PEER] | 262 | 2- 17 years old | *FLG*: massively parallel sequencing (introns and exons) | Persistence of AD: symptom-free time during 6 month periods | Moderate |
| Nomura 2013_[75]_ | Cohort study, Japan | 116 | Median age of 12 months [range 9 – 14 months] | 6 *FLG* mutations (R501X, 3321delA, S1695X, Q1701X, S2554X, S2889X, S3296X, K4022X) and single nucleotide polymorphisms (SNPs) near *FLG* (rs1933064, rs12730241) were genotyped | Presence of AD: paediatrician diagnosis, based on H+R criteria  Presence of food allergy: presence of food specific IgE > 0.35kU/L | Moderate |
| Palmer 2006_[76]_ | Three cohort studies:  1.Irish AD cohort  2. Scottish asthma cohort [BREATHE]  3. Danish birth cohort [COPSAC] | 1. AD children N =52, controls N =189  2. Asthmatic children N = 604, controls N = 1008  3. AD children N = 142, controls = 190 | Irish AD cohort: 'paediatric patients'  Scottish cohort: school children and adolescents  Danish cohort - recruited at birth, followed up during first 3 years of life | 2 *FLG* mutations: R501X and 2282Del4 | Irish Cohort  Presence of AD: dermatologist-diagnosed atopic dermatitis via UKWP criteria or H&R criteria  Scottish Cohort:  Presence of AD: parent reported questionnaire  Presence of asthma: diagnosis via Scottish Intercollegiate Guidelines / BTS guidelines  Danish Cohort  Development of AD from 0-3yo: clinical follow-up and diary cards  Development of asthma from 0-3yo: clinical review and diary cards | Good |
| Ruge 2021_[77]_ | Danish cohort study [COPSAC] | N=174 | Enrolled at birth, analysed from 0-3yrs | *FLG* “loss of function mutations – null vs wild type”, mutations not specified | Presence of AD from 0-3yo: diagnosed via H+R criteria  Severity of AD from 0-3yo: number of days with “active AD” | Good |
| Sasaki 2014_[78]_ | Japanese cohort study | 721 | 0 – 6yrs old | 8 *FLG* mutations: (p.R501*, c.3321delA, p.S1695*, p.Q1701*, p.S2554*, p.S2896*, p.S3296*, and p.K4022) | Presence of AD at 0-6yo: annual medical examinations, diagnosed via the Japanese Dermatological Association diagnostic criteria | Moderate |
| Savilahti, 2010 _[79]_ | Case-control study, Finland | Cases: 87  Control: 76 | Cases: mean 8.6yrs (range 8-9.1yrs)  Controls: mean 8.6yrs (range 8.1-9.3yrs) | 5 *FLG* null mutations: del22824, 501-C/T, R2447X, S3247, 3702delG | Presence of cow’s milk protein allergy (categorised into IgE-mediated and non-IgE mediated): positive oral food challenge +/- skin prick test to cow’s milk protein or elevated IgE to cow’s milk protein | Moderate |
| Schuttelaar 2009_[80]_ | Birth cohort [PIAMA], Netherlands | 934 | Recruitment at birth, analysed at 4 and 8 years old | 3 *FLG* mutations: R501X, 2282del4 and R2447X | Presence of AD at 4yo: ISAAC questionnaire  Presence of asthma at 0-8yo: parent-reported diagnosis and presence of symptoms in the previous 12-months  Presence of allergic rhinitis at 3-8yo: parent report | Good |
| Tan 2012_[81]]_ | Case-control - HealthNuts study (Australian population-based) | n = 700 one year old infants from the HealthNuts population based cohort study controls n = 126 | 1 year | 5 *FLG* mutations: R501X, 2282del4, R2447X, S3247X, and 3702delG | Presence of atopic dermatitis: parent-reported diagnosed AD or itchy rash (not nappy rash) treated with steroids, and/or nurse-observed current eczema during recruitment | Moderate |
| Vardar-Arcar_[82]_ | Case control, Turkey | N= 132 [FA], 273 [FA and AD], 61 [AD]  N=128 healthy controls | Food allergy median age 2yrs (IQR 1-5.5)  Food allergy and AD: median 1.0yrs (IQR 0.6-1.6)  AD only: median 0.3 yrs (IQR 0.1-1.6)  Controls: median 2.4yrs (IQR 1.4-3.5) | 4 *FLG* mutations R501X, 2282del4, R2447X and S3247X | Presence of AD: “chronic pruritus and eczematous dermatitis with typical age-specific morphology, distribution pattern, and a chronic relapsing course”  Severity of AD: SCORAD [categorised into severe or non-severe]  Presence of FA: “presence of a consistent clear cut history of allergic symptoms that occurred after the ingestion of a specific food”, with the either serum specific IgE >0.35 kU/L or a positive skin-prick test | Moderate |
| Venkataraman 2014_[83]_ | UK Cohort study [from IOW population based birth cohort study] | N= 1150 | 0 - 18yrs  Analysed at 1, 2, 4, 10 and 18 year time points] | 5 *FLG* mutations: R501X, 2282del4, S3247X, 3702delG, and R2447X | Presence of AD: via H&R criteria  Presence of food allergy: a positive skinprick test reaction to 1+ allergens tested | Good |
| Wan 2017_[84]_ | Cohort study, USA [from PEER study] | N= 798 | 2-17yrs old at enrolment | 4 *FLG* mutations [ R501X, 2282del3, R2447X, S3247X] | Age of onset of AD: into 0-6mo (3mo in analysis), 6-12mo (9mo in analysis) or greater than 12m old | Moderate |
| Wang 2011_[85]_ | Case control, Taiwan | N = 116 cases  N = 212 controls | AD group aged 2-5yrs old, aged matched controls [+/- 5 months] | 6 *FLG* common polymorphisms: T454A, P478S, E498D, H519N, R3270C, Q3322Q) and 10 rare mutations (R501X, S3247X, S3296X, S2554X, S2889X, 2282del4, 3321delA, E1795X, E2422X and Q2417X | Presence of AD: H+R criteria  Presence of asthma: ISAAC questionnaire  Presence of respiratory allergy: ISAAC questionnaire | Good |
| Wang 2015_[86]_ | Cohort study [Childhood Environmental and Allergic Diseases Cohort], Taiwan | N = 485 | 3 years old | *FLG* P478S (rs11584340) | Presence of AD: diagnosed by physicians as based on ISAAC questionnaire  Presence of asthma at 6yo: ISAAC questionnaire plus clinical criteria of:  1. asthma symptoms or asthma medications and a 12% increase in forced expiration volume in 1 second after the use of a bronchodilator  2. asthma symptoms or asthma medications and a history of asthma symptoms  3. history of asthma symptoms and wheeze at current examination  Presence of atopy: skinprick testing positive for 6 common allergens | Good |
| Weidinger 2008_[87]_ | Cohort [ISAAC phase II], Germany | N= 3099 | cohort mean age 9.6 (SD 0.6) | 5 *FLG* mutations: R501X, 2282del4, R2447X, S3247X, 3702delG | Presence of AD: physician-diagnosed, based on Atopic subcategory if positive skin prick testing against a1+ of the allergens tested  Current AD: defined as “presence of an itchy rash in the last 12 months that had affected the skin creases”  Presence of current wheeze  Atopic asthma: physician’s diagnosis of asthma (at least once) or of spastic bronchitis or recurrent asthmatic bronchitis (at least twice) in a self-administered questionnaire  Atopic subcategory if positive SPTs against at least 1 of the allergens tested  Presence of allergic rhinitis: diagnosis of hayfever by a clinician, in the presence of positive skin prick testing to 1+ allergens | Good |
| Yu 2013_[88]_ | Case control, South Korea | N = 1430 cases, N=862 controls | 0- 18yrs controls, mean age 9.47±3.37  AD mean age 5.17±3.82 | 3 *FLG* mutations: 3321delA, E2422X and R501X | Presence of AD: based on H&R criteria  Severity of AD: SCORAD. Atopic subcategory if as positive skin prick testing or presence of allergen specific IgE | Good |
| Ziyab 2012_[89]_ | UK Cohort (IOW birth cohort) | 1211 | recruited at birth analysed 3 transition periods 1-4/2-4y, 4-10y, 10-18y | 3 *FLG* mutations: R501X, 2282del4 and S3247X | Presence of AD at 1, 2, 4, 10 and 18yo: based on H&R criteria  Presence of respiratory allergy at 1 ad 2yo: positive skin prick testing to 1+ allergen | Good |
| Ziyab 2014_[90]_ | UK Cohort (IOW birth cohort) | 1456 | recruited at birth, followed up at 1, 2, 4, 10 and 18yo | *FLG* haploinsufficiency (for R501X, 2292del4 or S3247X) | Presence of AD at 1, 2, 4, 10 and 18yo: based on H&R criteria  Presence of asthma as: wheeze over the last 12 months and treatment given for asthma, asthma related symptoms at 1, 2 and 4yo or meeting ISAAC criteria at 10 and 18yo  Presence of food allergy: positive skin prick testing to 1+ of the tested food / aeroallergens  Presence of respiratory allergy: sneezing, or a runny or blocked nose without a cold or the flu in the last 12 months | Good |
| Ziyab 2018_[91]_ | UK Cohort studies  1. IOW birth cohort [n = 1456] 2. MAAS population based cohort study [n = 1058] | IOW = 1456 MAAS = 1058 | IOW cohort: recruited at birth – follow up at 1, 2, 4, 10 and 18 yrs  MAAS: recruited at birth – follow up aged 5, 8 and 11yrs | 3 *FLG* mutations: R501X, S3247X, 2282del4 | Presence of food allergy at 5, 8 and 11yo: positive skin prick testing to 1+ of tested food / aeroallergens | Good |
